# Stimuli Responsive Materials Supported by Orthogonal Hydrogen and Halogen Bonding or I···Alkene Interaction

**DOI:** 10.3390/molecules26247586

**Published:** 2021-12-14

**Authors:** Pierre Frangville, Shiv Kumar, Michel Gelbcke, Kristof Van Hecke, Franck Meyer

**Affiliations:** 1 Microbiology, Bioorganic and Macromolecular Chemistry Unit, Faculty of Pharmacy, Boulevard du Triomphe, 1050 Brussels, Belgium; Pierre.frangville@ulb.be (P.F.); shiv.kumar@ulb.be (S.K.); Michel.Gelbcke@ulb.be (M.G.); 2 XStruct, Department of Chemistry, Ghent University, Krijgslaan 281-S3, 9000 Ghent, Belgium; kristof.vanhecke@ugent.be

**Keywords:** halogen bonding, azobenzene, pH sensitive, stimuli responsive, orthogonal interaction

## Abstract

Smart materials represent an elegant class of (macro)-molecules endowed with the ability to react to chemical/physical changes in the environment. Herein, we prepared new photo responsive azobenzenes possessing halogen bond donor groups. The X-ray structures of two molecules highlight supramolecular organizations governed by unusual noncovalent bonds. In azo dye I-azo-NO_2_, the nitro group is engaged in orthogonal H···O···I halogen and hydrogen bonding, linking the units in parallel undulating chains. As far as compound I–azo–NH–MMA is concerned, a non-centrosymmetric pattern is formed due to a very rare I···π interaction involving the alkene group supplemented by hydrogen bonds. The Cambridge Structural Database contains only four structures showing the same I···CH_2_=C contact. For all compounds, an ^19^F-NMR spectroscopic analysis confirms the formation of halogen bonds in solution through a recognition process with chloride anion, and the reversible photo-responsiveness is demonstrated upon exposing a solution to UV light irradiation. Finally, the intermediate I–azo–NH_2_ also shows a pronounced color change due to pH variation. These azobenzenes are thereby attractive building blocks to design future multi-stimuli responsive materials for highly functional devices.

## 1. Introduction

Stimuli responsive materials are fascinating compounds with the ability to translate a stimulus into a change of physical/chemical properties at the nano/macro-scale [1]. The careful design of smart systems has opened new perspectives for the conception of next generation of highly functional materials in various domains [2,3]. The chemical toolbox is thereby comprised of a large array of molecular groups that can be finely modified/adapted for the creation of stimuli responsive drug delivery systems, sensors, smart surfaces or purification devices [4,5]. Typically, the responsivity of these compounds is triggered by varied stimuli such as a change of temperature [6], pH [7], magnetic environment [8], redox condition [9], photo-irradiation [10] and many others. In the arena of smart systems, photo-responsive materials have a photo switching ability in a reversible or irreversible fashion that consists in chemical bond breaking/formation or a conformational change upon irradiation [11]. Among these switchable compounds, azobenzenes represent a very appealing class of molecules that can reversibly isomerize from *trans* to *cis* form and vice versa [12]. An interesting feature lies in the fact that several synthetic pathways have been developed to prepare symmetric or asymmetric azobenzene derivatives [13]. Moreover, structural modifications of the azobenzene core allow for a fine tuning/shifting of absorbance in the red region and even the near-infrared, whereas shorter wavelength UV radiation is generally used to induce the isomerization [14,15].

In the last two decades, the halogen bonds (XB), i.e., the interactions involving a halogen as acceptor of electronic density, have become a huge point of interest in the scientific community [16,17]. Although similar in many aspects to hydrogen bonds, XB have demonstrated some specificities in synthetic chemistry [18], biological and materials sciences [19,20]. As far as the applications of azobenzene-based XB donors are concerned, the light induced surface patterning was achieved using asymmetric self-complementary azo dyes linked to poly(4-vinylpyridine) through N···I halogen bonds [21,22]. Other works have reported the formation of photomechanical azo crystals and a gelator capable of undergoing a gel-to-sol transition upon green light irradiation, involving symmetric XB compounds [23,24]. However, only a handful of instances has studied the design and application of such XB molecules, which are usually prepared in moderate yields [25]. Considering this, we decided to develop a straightforward access to photo responsive compounds with XB donor ability. In contrast to azo dyes already described in the literature, these compounds aim at being appended to a substrate such as a polymer which has never been described so far [26]. Recently, we have reported the preparation of stimuli-responsive polymers having the capability to react to a change of pH, magnetic field and UV-light irradiation [27]. In the same way, and as a preliminary result in the formation of new XB-based smart materials, our endeavors herein are devoted to the synthesis, X-ray structure analysis and determination of physico-chemical properties of new azo dyes endowed with XB donor capability, light responsiveness and even pH sensitivity for a synthetic intermediate.

## 2. Results & Discussion

### 2.1. Synthesis of Halogen Bonded Azobenzenes

**Scheme 1:**
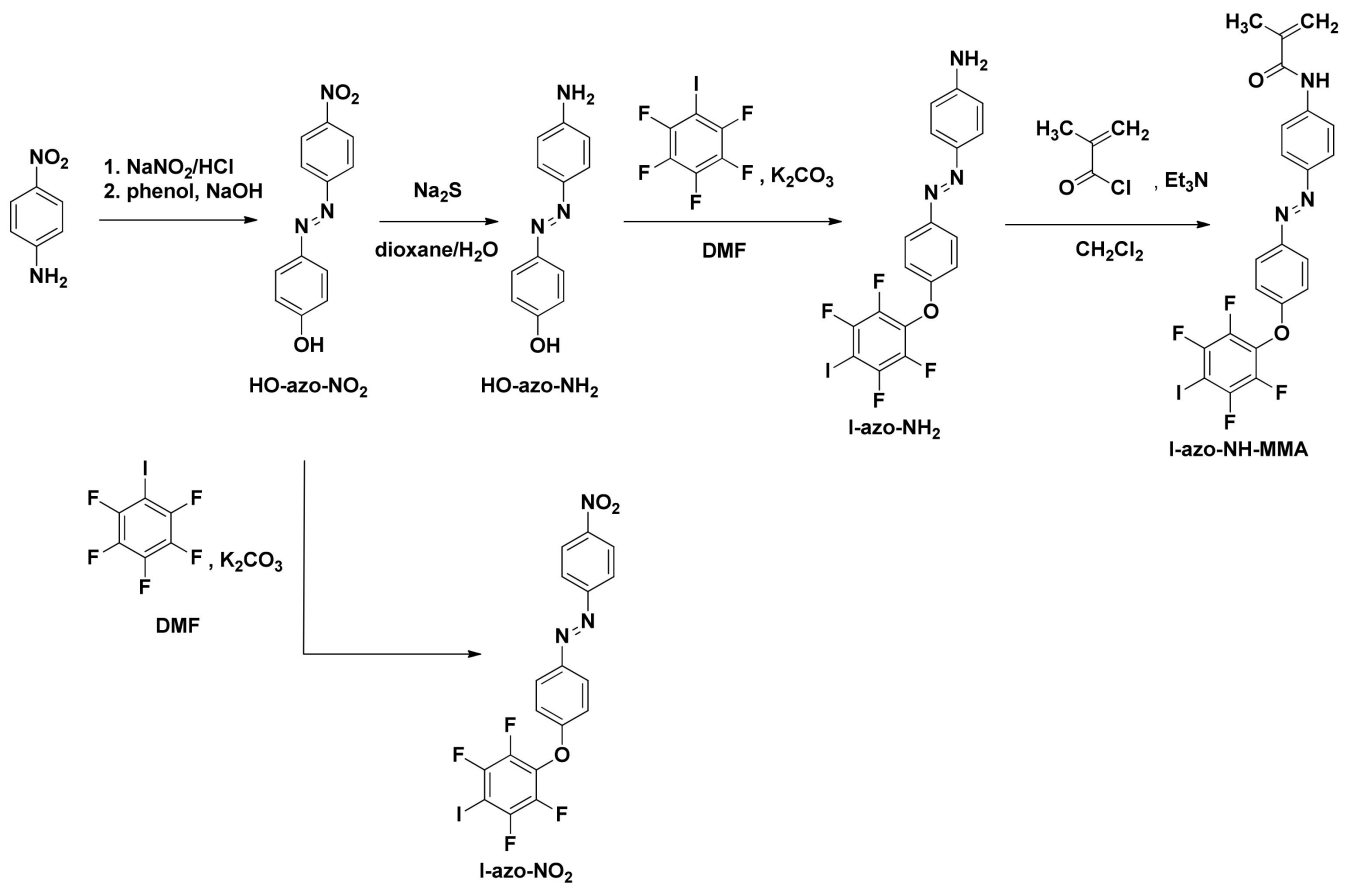
Synthesis of halogen bonded azobenzene derivatives.

The preparation of halogen bonded azobenzenes was performed through a multistep synthesis (Scheme 1). 

First of all, the azobenzene core, namely 4-((4-nitrophenyl)diazenyl)phenol (HO–azo–NO_2_), was obtained in 96% yield by diazotization of 4-nitroaniline with sodium nitrite in the presence of hydrochloric acid, followed by a coupling reaction with phenol in alkaline conditions, according to a procedure already described in the literature [28]. Subsequently, the incorporation of a halogen bond donor group was achieved by nucleophilic replacement of fluorine of pentafluoroiodobenzene using HO–azo–NO_2_ and potassium carbonate in dimethylformamide (DMF) at 50 °C. As such, the resulting I–azo–NO_2_ was isolated in 95% yield. In order to prepare a polymerizable azobenzene, HO–azo–NO_2_ was first reduced with a large excess of sodium sulfide (Na_2_S) in a dioxane/water mixture at 90 °C to give HO–azo–NH_2_ in 92% yield. Afterwards, the XB donor ring was introduced selectively on the hydroxy group to provide I–azo–NH_2_ in 91% yield. Finally, I–azo–NH–MMA was obtained by the reaction of methacryloyl chloride and I–azo–NH_2_ in the presence of triethylamine in dichloromethane at room temperature in 87% yield (Figures S3–S7, Supplementary Materials).

### 2.2. X-ray Structure Analysis of XB Azobenzenes 

Afterwards, the crystallographic structures of two azobenzene derivatives were studied as first evidence of their ability to form halogen bonds (Table 1).

**Figure 1:**
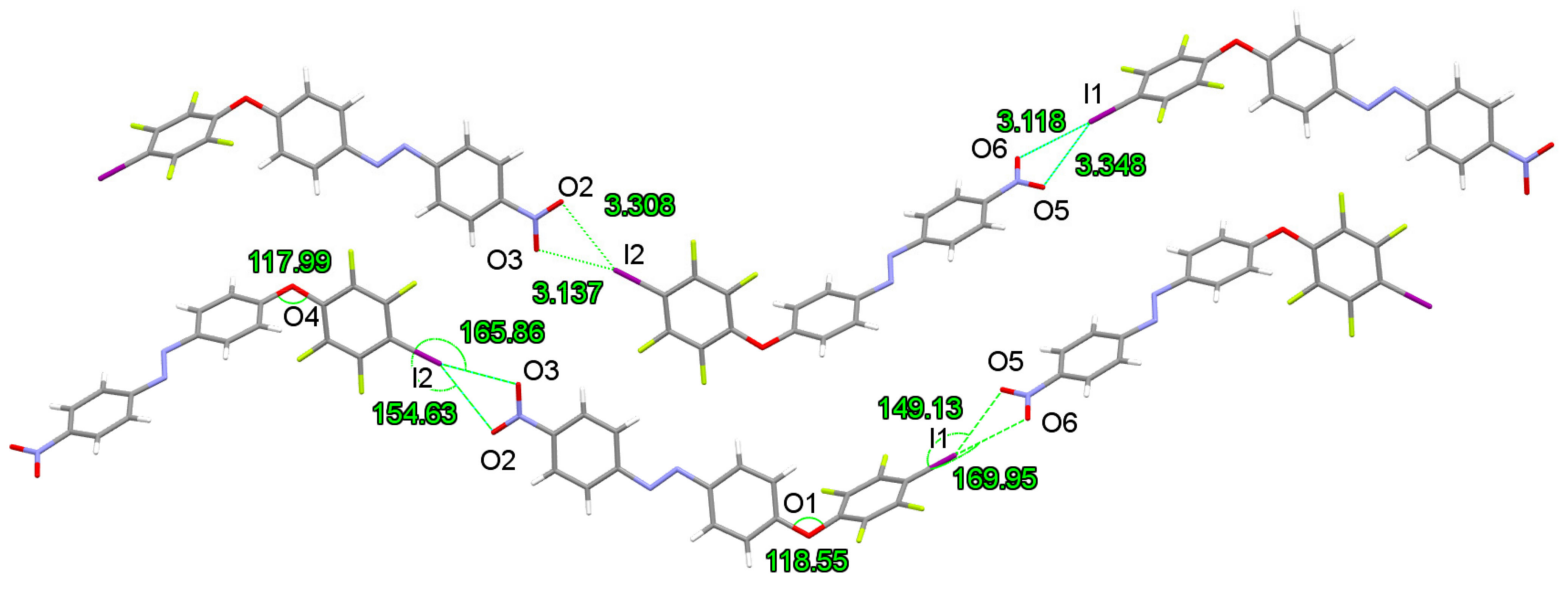
X-ray structure of I–azo–NO_2_ developing undulating parallel chains due to two O···I halogen bonds. Colors are as follows: grey, C; blue, N; red, O; white, H; yellow, F; purple, I. O···I bond distances (Å) and C–I···O bond angles (°).

The slow evaporation of a dichloromethane solution of I–azo–NO_2_ provided materials with a needle-like crystal morphology suitable for X-ray diffraction analysis. I–azo–NO_2_ crystallized in the centro-symmetric space group *P*-1, with the asymmetric unit consisting of two complete azobenzene molecules. From a general point of view, the self-complementary azobenzenes develop undulating parallel chains running in opposite directions due to non-covalent bonds (Figure 1).

The wavy character of this arrangement is induced by oxygen atoms linking the tetrafluoroiodobenzene group to the azobenzene core, showing dihedral angles of 63.0(1)° and 64.1(1)° between the tetrafluoroiodobenzene and benzene rings, for both molecules, respectively. NO_2_···I halogen bonds link the successive molecules into infinite chains and transfer the molecular information between almost equiplanar fluorinated and nitroaryl rings. In details, each iodine interacts with both oxygen atoms of the nitro group, but distances are slightly different, as defined in a Q-type interaction [29]. The I1···O5 and I2···O2 separations are 3.348(2) and 3.308(2) Å, respectively, i.e., around 5% shorter than the sum of van der Waals radii, whereas the I1···O6 and I2···O3 distances are slightly shorter (~90% of the sum of van der Waals radii) (Table 2) [30].

**Figure 2:**
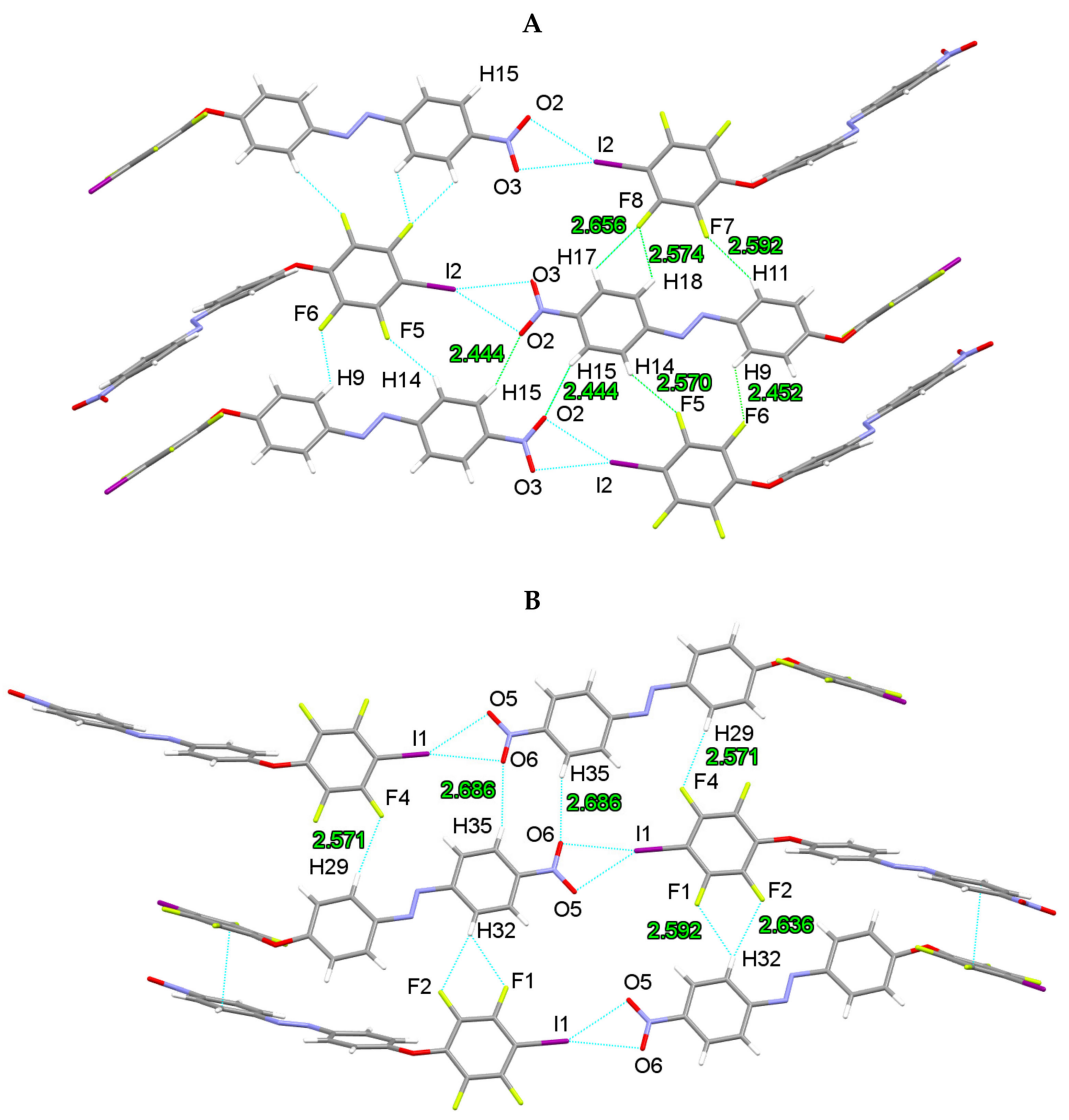
X-ray structure of I–azo–NO_2_ highlighting the H15···O2···I2 (**A**) and H35···O6···I1 (**B**) orthogonal hydrogen and halogen bonds.

In all cases, the C–I···O angles are in the range of ~149 to ~170°, which is consistent with halogen bonding parameters. Moreover, two adjacent units pointing in opposite directions are also connected through the nitro group by H15···O2 and H35···O6 hydrogen bonds of 2.444 and 2.686 Å, respectively. Therefore, O2 and O6 are simultaneously engaged in halogen and hydrogen bonds, the H15···O2···I2 and H35···O6···I1 angles being ~100.5 and ~93.7°, respectively (Figure 2). To date, few studies have reported an orthogonal relationship between these interactions [31]. Typically, such a supramolecular motif is observed in biological systems with a carbonyl group as electron donor [32]. Recently, a chloride anion was described as a hydrogen and halogen bond acceptor in a co-crystal composed of a pyrazolium salt and diiodotetrafluorobenzene [33]. It is interesting to note that the combination of these interactions also occurs in complexes of 4-nitrobenzamide/1,4-diiodobenzene and 1,4-dinitrobenzene/4-iodocinnamic acid [29,34]. Finally, the supramolecular scaffold is stabilized by a myriad of H···F contacts between head-to-tail adjacent molecules (Figure 2 and Table 2). The complementary bonded rings (i.e., tetrafluoroiodobenzene group and azobenzene core) belong to the same plane that generates a step-like organization.

**Figure 3:**
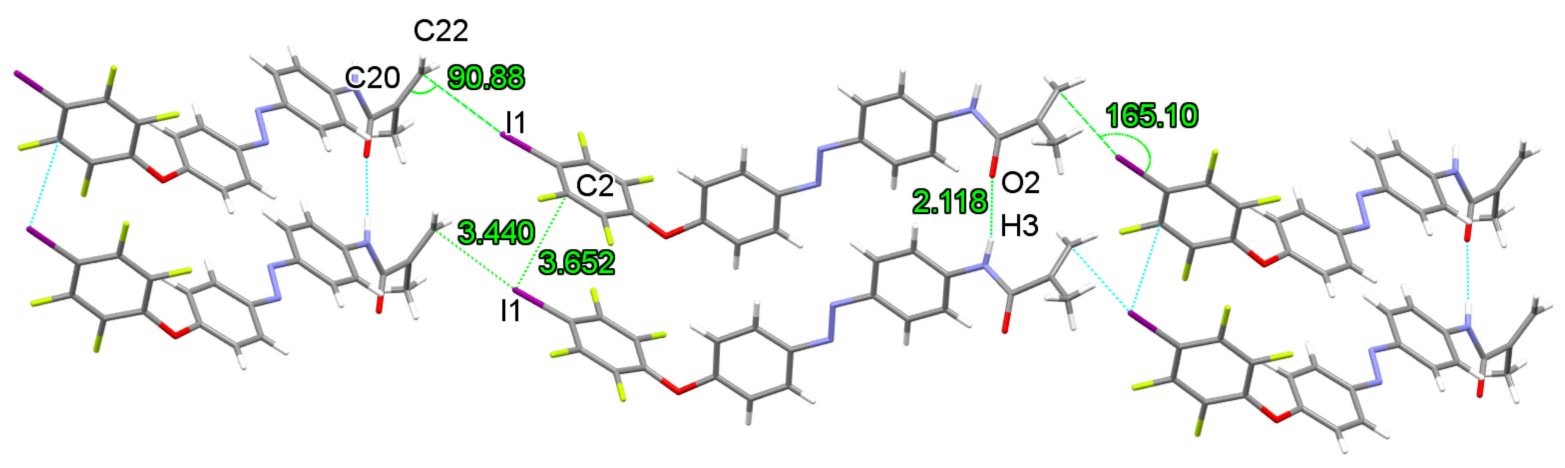
X-ray structure of I–azo–NH–MMA developing undulating parallel chains due to C···I halogen bonds.

Subsequently, the crystal formation of I–azo–NH–MMA was carried out using the aforementioned conditions. The compound crystallized in the non-centrosymmetric space group *P*na2_1_, with one azobenzene molecule in the asymmetric unit. The X-ray structure of the supramolecular scaffold adopts a similar undulating linear arrangement provoked by the tetrahedral geometry of oxygen with sp3 hybridization (Figure 3). Here, successive units are linked due to I···π halogen bonds involving the alkene functional group. The crystallographic parameters are in agreement with a halogen bond interaction, namely an I···C22 distance of 3.440(8) Å and a C–I···C22 angle close to linearity (165.1(3)°). Interestingly, the iodine atom makes a perpendicular approach to the double bond (C20–C22···I angle ~90°), emphasizing the electron donation from the p orbital.

This intriguing contact convinced us to make a Cambridge Structural Database (CSD version 5.42 updates, Sep 2021) analysis on the interaction between an iodine and a double bond (I···CH_2_=C, C···I distances between 3.0 and 4.0 Å). This survey reveals that only four molecules adopt the same structural motif, namely HOQKAC [35], HOTZOH [36], PEQVOZ [37] and USAJIK [38]. In these X-ray structures, the I···C interactions appear much weaker than in I–azo–NH–MMA, since the I···CH_2_=C distances are in the range of 3.526-3.692 Å (Figure S1, Supplementary Materials). In the XB-based azobenzene, a short distance exists between the negative equatorial region of the iodine atom and carbon atom of the electron deficient fluorinated group (I···C2 distance = 3.652 Å). This phenomenon highlights the dual character of halogen atoms, and more particularly of iodine, that can behave both as electron donor and acceptor [39,40]. Finally, the supramolecular architecture features N–H···O hydrogen bonds (H3···O2 distance of 2.12(6) Å) involving the amide functional group (graph set motif C(4)) [41]. Another interesting feature concerns the layered organization of the units in a non-centrosymmetric fashion. Further studies will concern the non-linear optical properties of this compound [42,43].

### 2.3. Halogen Bonding Properties

The crystallographic study of I–azo–NO_2_ and I–azo–NH–MMA has confirmed that the tetrafluoroiodobenzene ring is a versatile and reliable XB donor group allowing the formation of complicated architectures by self-assembly [44].

**Figure 4:**
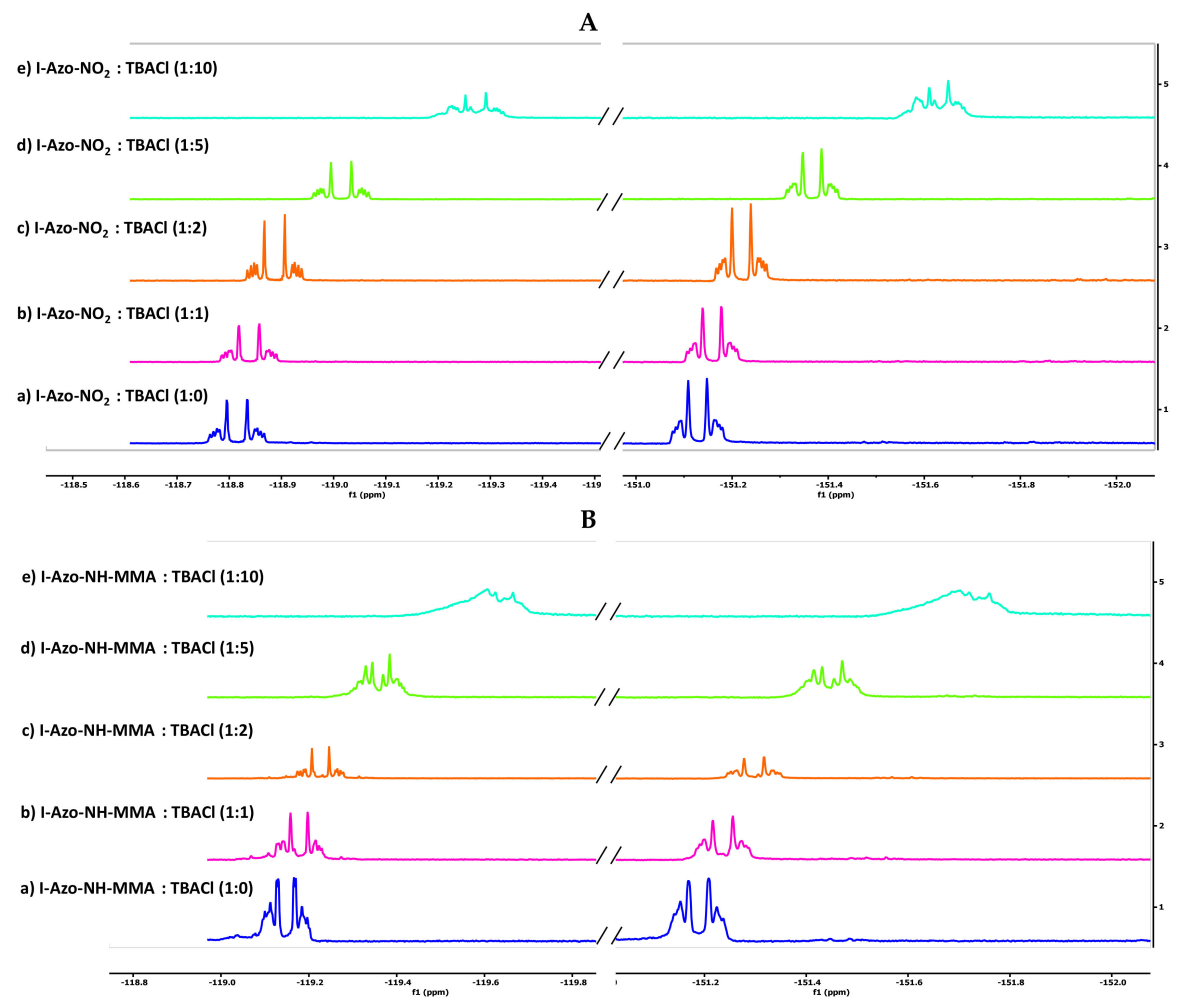
Stack plot of ^19^F-NMR spectra of I–azo–NO2 and I–azo–NH–MMA (CDCl_3_), at different molar ratios of I-azo-NO_2_/TBACl (**A**) and I–azo–NH–MMA/TBACl (**B**). Molar ratios: a) 1:0; b) 1:1; c) 1:2; d) 1:5; e) 1:10.

A further study aimed at highlighting the ability of the XB group to function in an intermolecular recognition process. Halide anions are particularly effective in the construction of robust supramolecular architectures, and several research works have reported the sensing of anions by halogen bonding [45,46]. Therefore, an ^19^F-NMR spectroscopic analysis was performed to monitor the interaction of the XB donor azo dyes with chloride anions in solution. In practice, I–azo–NO_2_ and I–azo–NH–MMA were challenged with increasing quantities of tetrabutylammonium chloride (TBACl) in deuterated chloroform using bis(2,2,2-trifluoroethyl) ether as internal reference standard. The NMR titration experiments reveal an upfield shift of both C–F signals corresponding to fluorine atoms *ortho* and *meta* to the iodine atom upon treatment with TBACl (Figure 4). The ^19^F-NMR resonances for I–azo–NO_2_ and I–azo–NH–MMA exhibit marked variations with *δ* decreasing by ca. −0.5 ppm in the presence of 10 equivalents of TBACl. Note that the same trend is observed with I–azo–NH_2_ (Figure S2, Supplementary Materials). In these cases, this shifting behavior is attributed to formation of I···Cl¯ halogen bonded adducts in solution (Figure S8, Supplementary Materials).

### 2.4. Photo-Responsive Properties

**Figure 5:**
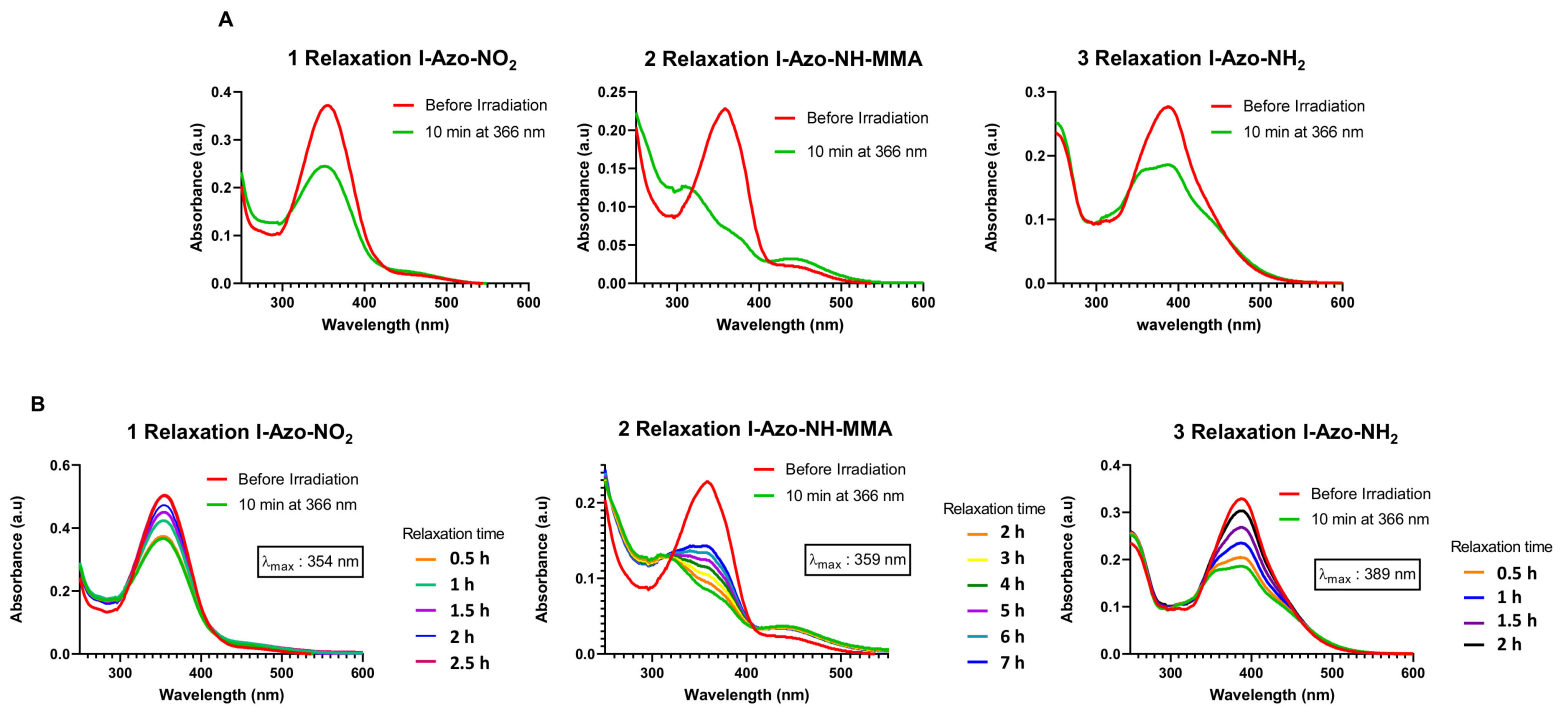
UV-Vis spectra for I–azo–NO_2_ (**A1**), I–azo–NH–MMA (**A2**) and I–azo–NH_2_ (**A3**) before (red) and after (green) a 10 min exposure to 366 nm light. Thermal relaxation spectra in the dark for I–azo–NO_2_ (**B1**), I–azo–NH–MMA (**B2**) and I–azo–NH2 (**B3**).

Subsequently, the photo-responsiveness of these compounds was investigated. For this, dichloromethane solutions of I–azo–NO_2_ and I–azo–NH–MMA and an acetonitrile solution of I–azo–NH_2_ were treated by UV light irradiation (366 nm) (Figure 5). As concerns I–azo–NO_2_ and I–azo–NH–MMA, the UV-Vis spectra reveal a significant decrease of the main band at ~360 nm assigned to the π→π* absorption, and a slight strengthening of n→π* band at ~460 nm. The same trend is observed for I–azo–NH_2_ but the main band is slightly shifted at 389 nm. These variations are typically observed for a conformational change of the diazobenzene unit, indicating a *trans* to *cis* isomerization. The reversible character of this phenomenon was demonstrated through a relaxation kinetic study that occurs within 8 h for all compounds after leaving the solutions in darkness or after irradiation with white light for 2 min (Figure 5).

### 2.5. pH Sensitivity

**Figure 6:**
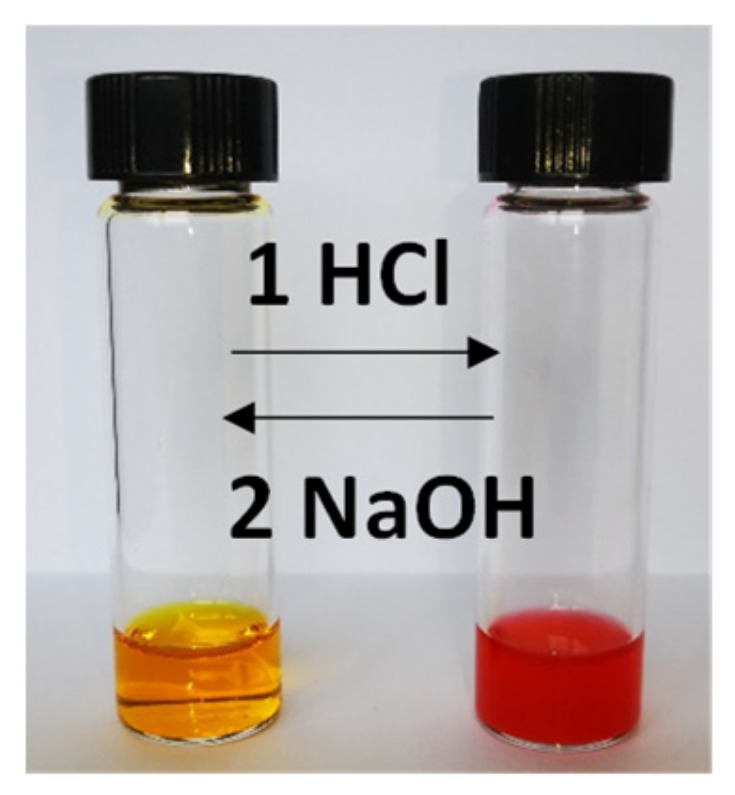
Acetonitrile solution of I–azo–NH2 (left) and after acidic treatment (right).

4-aminoazobenzene belongs to a family of dyes that undergo protonation accompanying a pronounced color change in acidic environment [47]. In addition to the isomerization and XB donor properties, the pH sensitivity of I–azo–NH2 was demonstrated in solution. After solubilization in acetonitrile, the neutral medium reveals an orange color that turns red after addition of one drop of concentrated HCl 36% (Figure 6). Afterwards, this system exhibits a reversible color change upon addition of a few drops of sodium hydroxide. I–azo–NH2 is thereby endowed with a triple stimuli responsiveness. 

## 3. Materials and Methods

All non-aqueous reactions were run in oven-dried glassware under a positive pressure of argon or nitrogen, with exclusion of moisture from reagents and glassware using standard techniques for manipulating air-sensitive compounds. Solvents were purchased from Chemlabs (Zedelgem, Belgium) and dried using molecular sieves of 4 Å or standard distillation techniques. Iodopentafluorobenzene was purchased from Fluorochem (Derbyshire, UK). Nitroaniline was purchased from Apollo scientific (Bredbury, Stockport, UK) (nitroaniline was purified by flash chromatography on silica gel before use). Na_2_S, NaNO_2_ and phenol were purchased from Merck (Frankfurt, Germany). Methyl methacrylate was purchased from TCI chemicals (Zwijndrecht, Belgium). Reactions were monitored by analytical thin-layer chromatography (TLC) performed on pre-coated, aluminum-backed silica gel plates. Visualization of the developed chromatogram was performed by UV absorbance at 254 nm or 366 nm. Flash chromatography was performed on a 230–400 mesh silica gel with the indicated solvent systems. Infrared spectra were recorded on an IRAffinity-1 Shimadzu (Duisburg, Germany) FT-IR spectrometer and are reported in reciprocal centimeters (cm^−1^). Routine nuclear magnetic resonance spectra were recorded on a JEOL 400 MHz (Zaventem, Belgium). Chemical shifts for 1H and 13C-NMR spectra are recorded in parts per million from solvent resonance as the internal standard. Data are reported as follows: chemical shift, integration, multiplicity (s = singlet, d = doublet, dd = double doublet, t = triplet, q = quartet, m = multiplet and br = broad), and coupling constants are in Hz. High-resolution mass spectrometry spectra were recorded on a MS QTOF-6520 Agilent (Santa Clara, CA, USA) for structure confirmation. UV Spectra were recorded on a Spectrophotometer Shimadzu (Duisburg, Germany) UV-1800 in the 250 nm–600 nm range for each spectrum.

### 3.1. Synthetic Procedures

*4-((4-nitrophenyl)diazenyl)phenol (HO-azo-NO_2_)*. This compound was prepared according to a previously reported procedure [28]. 4-Nitroaniline (3 g, 0.022 mol) was added to the hydrochloric acid aqueous solution (10%, 50 mL) and was stirred until complete dissolution. Sodium nitrite (1.95 g, 0.029 mol) was dissolved in water (40 mL) and was added dropwise to the above solution within 1 h, then stirred for another 30 min, keeping the temperature below 0 °C. Phenol (2.66 g, 0.028 mol) and sodium hydroxide (1.11 g, 0.028 mol) were dissolved in water (40 mL), and the mixture was added dropwise to the diazonium salt solution within 1 h, keeping the temperature below 0 °C. The reaction was completed after stirring for another 3 h below 2 °C. After stirring at room temperature for 24 h, an orange product was precipitated and collected by filtration to afford HO–azo–NO_2_. Yield: 96%. Mp: 218–220 °C. ^1^H-NMR (400 MHz, CDCl_3_, ppm) δ 8.39 (2H, d, *J* = 9.2 Hz), 8.00 (2H, d, *J* = 9.2 Hz), 7.96 (2H, d, *J* = 8.8 Hz), 7.00 (2H, d, *J* = 8.8 Hz), 5.59 (1H, br). ^13^C-NMR (400 MHz, CDCl_3_, ppm) δ, 160.42, 156.16, 148.36, 147.01, 126.01, 124.86, 123.24, 116.27.

*4-((4-(2,3,5,6-tetrafluoro-4-iodophenoxy)phenyl)diazenyl)nitrobenzene (I-azo-NO_2_)*. HO–azo–NO_2_ (1.0 g, 1 equiv.) was dissolved in DMF (15 mL) at 50 °C. Potassium carbonate (1.3 equiv.) was added to the reaction mixture and vigorously stirred for 20 min. Pentafluoroiodobenzene (1.1 equiv.) was added dropwise to the reaction mixture and the reaction was stirred for 6 h. Upon complete conversion of HO–azo–NO_2_, the reaction was precipitated in water and the solid was washed with *n*-hexane yielding I–azo–NO_2_ as a deep red solid. Furthermore, the aqueous layer was extracted with diethyl ether three times, and the organic layer was dried over anhydrous sodium sulphate (Na_2_SO_4_) and concentrated to afford I-azo-NO_2_. Yield: 95%. Mp: 165–168 °C. ^1^H-NMR (400 MHz, CDCl_3_, ppm) δ 8.37 (2H, d, *J* = 9.2 Hz), 8.00 (4H, m), 7.13 (2H, d, *J* = 9.2 Hz,). ^13^C-NMR (101 MHz, CDCl_3_, ppm) δ 159.78, 155.79, 148.94, 148.82, 125.69, 124.89, 123.53, 116.26. ^19^F-NMR (376 MHz, CDCl_3_, ppm) δ −118.83, −151.14. HR-MS (ESI) [M − H]^+^ Calculated: 517.9619; Found: 517.9618 (0.26 ppm)

*4-((4-aminophenyl)diazenyl)phenol (HO-azo-NH_2_)*. This compound was prepared according to a previously reported procedure [28]. HO–azo–NO_2_ (3 g, 1 equiv., 12.35 mmol) is dissolved in dioxane (50 mL) and an aqueous solution of Na_2_S (3.85 g, 6 equiv., 49.4 mmol) is added to the reaction mixture at 70 °C in two times t = 0 min and t = 3 h The reaction is stirred overnight and stopped once there is no more evidence of starting material checked by TLC. Dioxane is concentrated and the crude material is extracted three times with diethyl ether, washed with an aqueous solution of sodium carbonate, dried over sodium sulphate and the organic layer is concentrated affording a red solid. The crude material was purified by column chromatography using dichloromethane as eluent to afford HO–azo–NH_2_. Yield: 92%. Mp: 185 °C (decomposed). ^1^H-NMR (400 MHz, CD_3_OD, ppm) δ 7.68 (2H, d, *J* = 8.8 Hz), 7.65 (2H, d, *J* = 8.8 Hz), 6.86 (2H, d, *J* = 8.8 Hz), 6.74 (2H, d, *J* = 8.8 Hz). ^13^C-NMR (101 MHz, CD_3_OD, ppm) δ 160.66, 152.53, 147.75, 145.86, 125.48, 124.94, 116.56, 115.37. 

*4-((4-(2,3,5,6-tetrafluoro-4-iodophenoxy)phenyl)diazenyl)aniline (I–azo–NH_2_)*. HO-azo-NH_2_ (2.15 g, 1 equiv., 10.10 mmol) was dissolved in DMF (25 mL) at 50 °C. Potassium carbonate (1.81 g, 1.3 equiv., 13.13 mmol) was added to the reaction mixture and vigorously stirred for 20 min. Pentafluoroiodobenzene (2.97 g, 1.2 equiv., 20.2 mmol) was added dropwise to the reaction mixture and the reaction was stirred for 6 h. Upon complete conversion of HO–azo–NH_2_, the reaction was precipitated in water and the solid was washed with *n*-hexane yielding I–azo–NH_2_ as an orange solid. Furthermore, the aqueous layer was extracted with diethyl ether three times, and the organic layer was dried over anhydrous Na_2_SO_4_ and concentrated to afford I–azo–NH_2_. Yield: 91%. Mp: 165–168 °C. ^1^H-NMR (400 MHz, CDCl_3_, ppm) δ 7.85 (2H, d, *J* = 9.2 Hz), 7.78 (2H, d, *J* = 8.8 Hz), 7.06 (2H, d, *J* = 9.2 Hz), 6.73 (2H, d, *J* = 8.8 Hz), 4.05 (2H, br). ^13^C-NMR (101 MHz, CDCl_3_, ppm) δ 157.98, 149.72, 149.52, 145.58, 125.20, 124.22, 116.07, 114.77. ^19^F-NMR (376 MHz, CDCl_3_, ppm) δ −119.35, −151.26. HR-MS (ESI) [M + H]^+^ Calculated: 487.9877; Found: 487.9900 (4.54 ppm)

*N-(4-((4-(2,3,5,6-tetrafluoro-4-iodophenoxy)phenyl)diazenyl)phenyl)methacrylamide (I–azo–NH–MMA)*. To a stirred solution of I–azo–NH_2_ (0.5 g, 1.03 mmol, 1 equiv.) in dichloromethane (3 mL) were added triethylamine (430 µL, 3.08 mmol, 3 equiv.) and methacryloyl chloride (150 µL, 1.54 mmol, 1.5 equiv.) at 0 °C. After stirring for 5 min, the mixture was allowed to warm up to room temperature and stirred for 1 h. Then the reaction was quenched with H_2_O and extracted with dichloromethane (3 × 15 mL). The combined organic layers were washed with brine, dried over anhydrous Na_2_SO_4_, concentrated under reduced pressure and purified by column chromatography using dichloromethane as eluent to afford I–azo–NH–MMA. Yield: 87%. Mp: 200–202 °C. ^1^H-NMR (400 MHz, CDCl_3_) δ 7.93 (2H, d, *J* = 8.8 Hz), 7.929 (2H, d, *J* = 9.2 Hz) 7.74 (2H, d, *J* = 9.2 Hz), 7.67 (s, 1H), 7.09 (2H, d, *J* = 8.8 Hz), 5.84 (1H, m), 5.52 (1H, m), 2.09 (3H, dd, *J* = 1.6 Hz, *J* = 0.9 Hz). ^13^C-NMR (101 MHz, CDCl_3_) δ 166.66, 158.72, 149.07, 149.02, 140.96, 140.58, 124.83, 124.24, 120.45, 120.10, 116.14, 18.88. ^19^F-NMR (376 MHz, CDCl_3_) δ −119.17, −151.21. HR-MS (ESI) [M + H]^+^ Calculated: 556.0150; Found: 556.0149 (1.65 ppm)

### 3.2. X-ray Crystallography

For the structures of I–azo–NO_2_ and I–azo–NH–MMA, X-ray intensity data were collected at 100 K, on a Rigaku Oxford Diffraction (Rigaku Corporation, Oxford, UK) Supernova Dual Source (Cu at zero) diffractometer equipped with an Atlas CCD detector using ω scans and CuKα (λ = 1.54184 Å) radiation. The images were interpreted and integrated with the program CrysAlisPro (versions 1.171.40.53 and 1.171.40.67a; Rigaku Corporation, Oxford, UK) [48]. Using Olex2, [49] the structures were solved by direct methods using the ShelXT structure solution program and refined by full-matrix least-squares on F^2^ using the ShelXL program package [50,51]. Non-hydrogen atoms were anisotropically refined and the hydrogen atoms in the riding mode with isotropic temperature factors fixed at 1.2 times U(eq) of the parent atoms (1.5 times for methyl groups). For I–azo–NH–MMA, the amide H-atom was located from a difference Fourier electron density map and refined with an N–H restrained distance of 0.88 Å.

### 3.3. ^19^F-NMR Titrations

Titrations of I–azo–NO_2_, I–azo–NH_2_ and I–azo–NH–MMA with the tetrabutylammonium chloride salt (TBACl) were monitored by ^19^F-NMR. Stock solutions of 25 mM (2 mL) and 100 mM (1 mL) of the azo dyes and TBACl were prepared in CDCl_3_. Bis(2,2,2-trifluoroethyl) ether was used as internal standard. Titration samples were prepared as follows (Table 3):

### 3.4. UV–Vis Analysis of Azobenzenes

UV-visible spectroscopic studies were performed with solutions of 10 µM of azobenzene derivatives in dichloromethane for I–azo–NO_2_ and I–azo–NH–MMA and acetonitrile for I–azo–NH_2_. Spectra were recorded before and after irradiation at 366 nm for 10 min. The kinetics of *cis* to *trans* isomerization were determined by recording absorption spectra every 30 min for 8 h after 10 min of irradiation at 366 nm.

### 3.5. pH Sensitivity

3 mg of I–azo–NH_2_ were solubilized in 3 mL of acetonitrile. After addition of two to three drops of HCl 36%, the solution turned red. Then, 2 drops of concentrated NaOH solution (28 M) were added, leading to an orange solution.

## 4. Conclusions

In summary, we have reported the synthesis of new azobenzene derivatives bearing halogen bond donor groups. The X-ray analyses of I–azo–NO_2_ and I–azo–NH–MMA highlight supramolecular arrangements developing undulating chains due to non-covalent bonds. In I–azo–NO_2_, the nitro group is simultaneously engaged in orthogonal hydrogen and halogen bonds, which has been scarcely reported to date. In light of the literature, the self-complementary Ar–I···O_2_N–Ar synthon can self-assemble in a head-to-tail mode, arising thus as a very appealing motif to design supramolecular architectures in a high reliable fashion (Scheme S1, Supplementary Materials). Regarding I–azo–NH–MMA, a non-centrosymmetric organization was observed thanks to uncommon I···CH_2_=C halogen bonding. A survey of the Cambridge Structural Database reveals that only four structures involve this contact. In addition to the crystallographic studies, ^19^F-NMR spectroscopic analyses have emphasized that such compounds can be involved in intermolecular recognition processes, as proposed through I···Cl^−^ halogen bonding in solution. Subsequently, the photo-responsiveness of all XB-based compounds was studied by irradiation at 366 nm. As expected, a *trans* to *cis* isomerization was confirmed by UV-Vis analysis, this phenomenon being reversible. Finally, the synthetic intermediate I–azo–NH_2_ shows a light and pH responsive profile in combination with a halogen bond donor ability, with a potential application in anion sensing. Further works will aim at preparing polymeric materials using these stimuli responsive units and their application in the preparation of functional compounds.

## Figures and Tables

**Table 1:** X-ray crystal data and refinement parameters for structures I–azo–NO_2_ and I–azo–NH–MMA.

	I–azo–NO_2_	I-azo–NH–MMA
Molecular formula	C_18_H_8_F_4_IN_3_O_3_	C_22_H_14_F_4_IN_3_O_2_
Formula weight (g·mol^−1^)	517.17	555.26
*T* (K)	100(2)	100(2)
Crystal system	triclinic	orthorhombic
Space group	*P*-1 (No. 2)	*P*na2_1_ (No. 33)
a (Å)	5.65780(10)	11.1393(2)
b (Å)	9.9399(2)	5.07170(10)
c (Å)	32.4971(4)	36.6524(7)
α (°)	91.9240(10)	90
β (°)	91.6640(10)	90
γ (°)	105.8040(10)	90
V (Å^3^)	1756.11(5)	2070.68(7)
*Z*	4	4
ρ_calc_ (g·cm^−3^)	1.956	1.781
μ (mm^−1^)	14.975	12.712
F (000)	1000	1088
2θ_max_ (°)	147.59	147.34
Measured reflections	61712	7384
Unique reflections	6933	3466
Observed reflections (*I* > 2σ(*I*))	6250	3128
Parameters refined	523	293
*R*_1_	0.0250	0.0428
*wR*_2_	0.0559	0.1109
*R*_1_ (all data)	0.0302	0.0485
*wR*_2_ (all data)	0.0584	0.1179
Goodness-of-fit (GOF)	1.030	1.054
CCDC-entry	CCDC 2117642	CCDC 2117643

**Table 2:** Parameters of halogen and hydrogen bonds in I–azo–NO_2_**.**

d (I···O), Å	d (I···O)/ΣvdWr, %	< (C–I···O), (H···O···I), °	d (H···O), Å	d (H···F), Å
3.348(2) (I1···O5)	~4.5	149.13 (C–I1···O5)	2.686 (H35···O6)	2.571 (H29···F4)
3.118(2) (I1···O6)	~11	169.95 (C–I1···O6)	2.444 (H15···O2)	2.592 (H32···F1)
3.308(2) (I2···O2)	~5.5	154.63 (C–I2···O2)		2.636 (H32···F2)
3.137(2) (I2···O3)	~10.5	165.86 (C–I2···O3)		2.656 (H17···F8)
		100.54 (H15···O2···I2)		2.574 (H18···F8)
		93.68 (H35···O6···I1)		2.592 (H11···F7)
				2.570 (H14···F5)
				2.452 (H9···F6)

**Table 3:** ^19^F-NMR titration of azo dyes with tetrabutylammonium chloride salt.

Molar Ratio of Azo-Dye:TBACl	Volume of Azo-Dye Stock Solution (µL)	Volume of TBACl Stock Solution (µL)	Volume of CDCl_3_ (µL)	Total Volume (µL)
1:0	200	0	500	700
1:1	200	50	450	700
1:2	200	100	400	700
1:5	200	250	250	700
1:10	200	500	0	700

## Data Availability

The data presented in this study are available in the Supplementary Materials.

## Supplementary Materials

Figure S1: X-ray structures of HOQKAC (**A**), HOTZOH (**B**), PEQVOZ (**C**) and USAJIK (**D**) showing the I···CH_2_=C halogen bonds found in the Cambridge Structural Database (CSD version 5.42 updates, Sep 2021). Colors are as follows: grey, C; blue, N; red, oxygen; white, H; orange, S; purple, I. Figure S2: Stack plot of ^19^F-NMR spectra of I–azo–NH_2_ (CDCl_3_), at different molar ratios of I–azo–NH_2_/TBACl. Molar ratios: (a) 1:0; (b) 1:1; (c) 1:2; (d) 1:5; (e) 1:10. Figure S3. ^1^H and ^13^C-NMR spectra for compound OH–azo–NH_2_. Figure S4. ^1^H and ^13^C-NMR spectra for compound OH–azo–NO_2_. Figure S5. ^1^H, ^19^F and ^13^C-NMR spectra for compound I–azo–NO_2_. Figure S6. ^1^H, ^19^F and ^13^C-NMR spectra for compound I–azo–NH_2_. Figure S7. ^1^H, ^19^F and ^13^C-NMR spectra for compound I–azo–NH–MMA. Scheme S1. Self-complementary Ar–I···O_2_N–Ar synthon. Halogen and hydrogen bonds are represented in blue and red dashed lines, respectively. Figure S8. ^19^F NMR titration curves of I–azo–NO_2_ (**A**), I–azo–NH_2_ (**B**) and I–azo–NH–MMA (**C**) with tetrabutylammonium chloride. Molar ratios (azo dyes/TBACl): 1:0; 1:1; 1:2; 1:5; 1:10.
