# Supplementary material for: Stimuli Responsive Materials Supported by Orthogonal Hydrogen and Halogen Bonding or I···Alkene Interaction
Source: Molecules. 2021 Dec 14;26(24):7586. doi: 10.3390/molecules26247586 (PMC8709106; doi:10.3390/molecules26247586)
Supplement: Supplementary file 1 [file molecules-26-07586-s001.zip › molecules-1457724-supplementary.pdf]

molecules

Supplementary Materials:

Stimuli responsive materials supported by orthogonal hydrogen and halogen bonding or I···alkene interaction

Pierre Frangville,[1] Shiv Kumar, [1] Michel Gelbcke,[1] Kristof Van Hecke,[2] and Franck Meyer [1,*]

[1] Microbiology, Bioorganic and Macromolecular Chemistry Unit, Faculty of Pharmacy, Boulevard du Triomphe, 1050 Brussels, Belgium; franck.meyer@ulb.be
[2] XStruct, Department of Chemistry, Ghent University, Krijgslaan 281-S3, B-9000 Ghent, Belgium; kristof.vanhecke@ugent.be
* Correspondence: franck.meyer@ulb.be; Tel.: +32-(0)2-650 51 96

Table of contents

X ray structures of compounds showing the I···CH_2=C halogen bonds found in the Cambridge Structural Data base 2

Stack plot of ^{19}F NMR spectra at different molar ratios of I–azo–NH2/TBACl 2

^{1}H ^{19}F and ^{13}C NMR spectra for all compounds 3

Scheme representing the self-complementary Ar–I···O_2N–Ar synthon 11

NMR titration of azo dyes with tetrabutylammonium chloride 11

X ray structures of compounds showing the I···CH_2=C halogen bonds found in the Cambridge Structural Data base

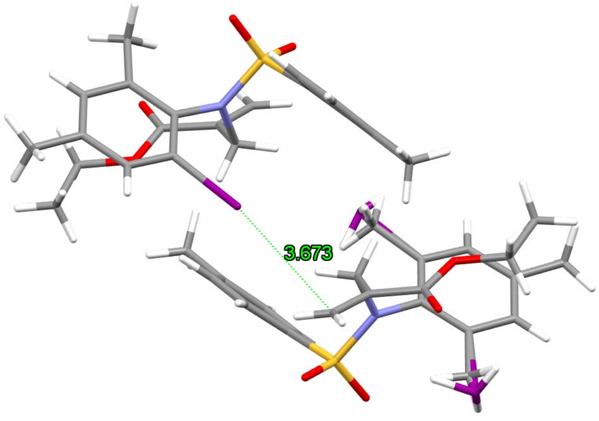

Copyright: © 2021 by the authors. Licensee MDPI, Basel, Switzerland. This article is an open access article distributed under the terms and conditions of the Creative Commons Attribution (CC BY) license (https://creativecommons.org/licenses/by/4.0/).

A

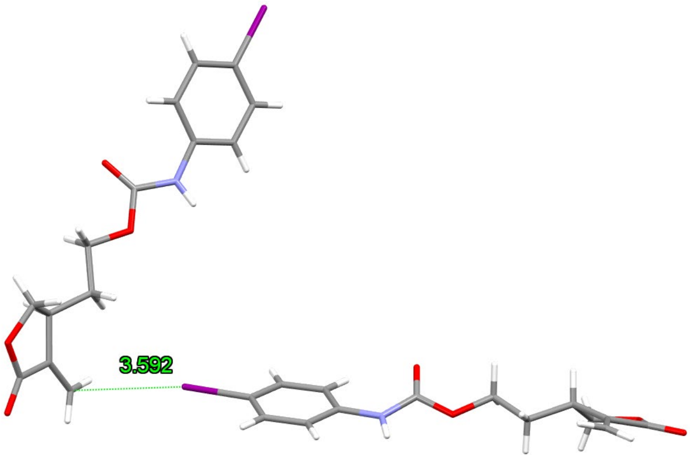

B

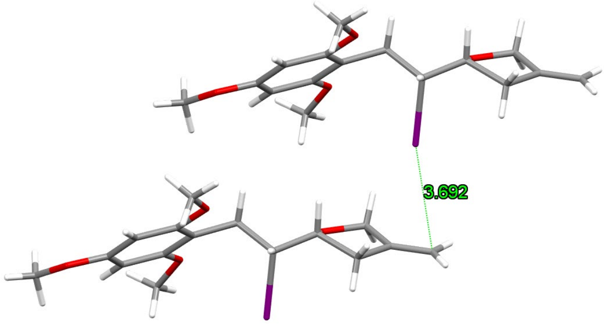

C

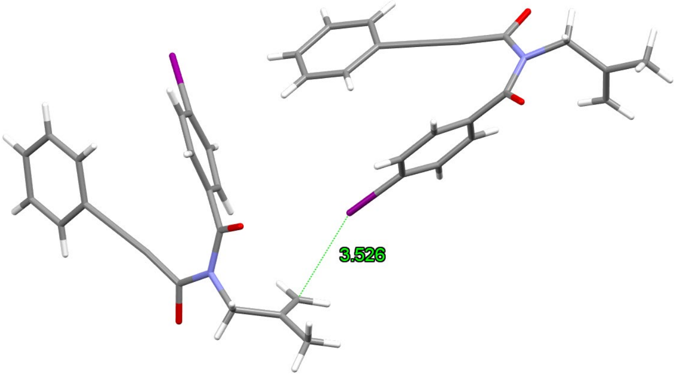

D

Figure S1. X ray structures of HOQKAC (**A**), HOTZOH (**B**), PEQVOZ (**C**) and USAJIK (**D**) showing the I···CH_2=C halogen bonds found in the Cambridge Structural Data base (CSD version 5.42 updates, Sep 2021). Colors are as follows: grey, C; blue, N; red, oxygen; white, H; orange, S; purple, I.

Stack plot of ^{19}F NMR spectra at different molar ratios of I–azo–NH2/TBACl

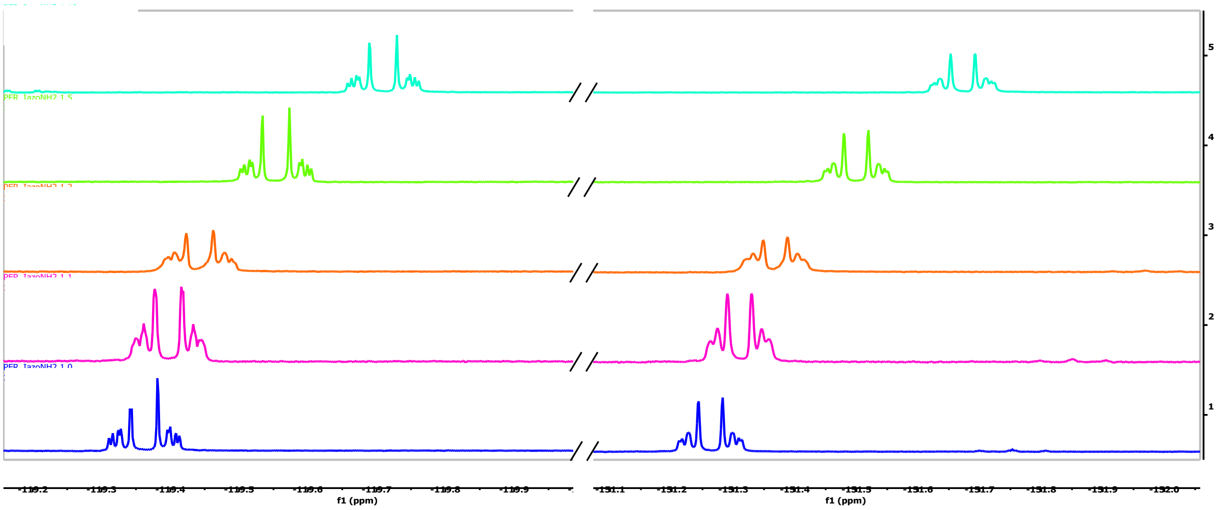

Figure S2. Stack plot of ^{19}F-NMR spectra of I–azo–NH_2 ($CDCl_3$), at different molar ratios of I–azo–NH_2/TBACl. Molar ratios: a) 1:0; b) 1:1; c) 1:2; d) 1:5; e) 1:10.

^{1}H ^{19}F and ^{13}C-NMR spectra for all compounds

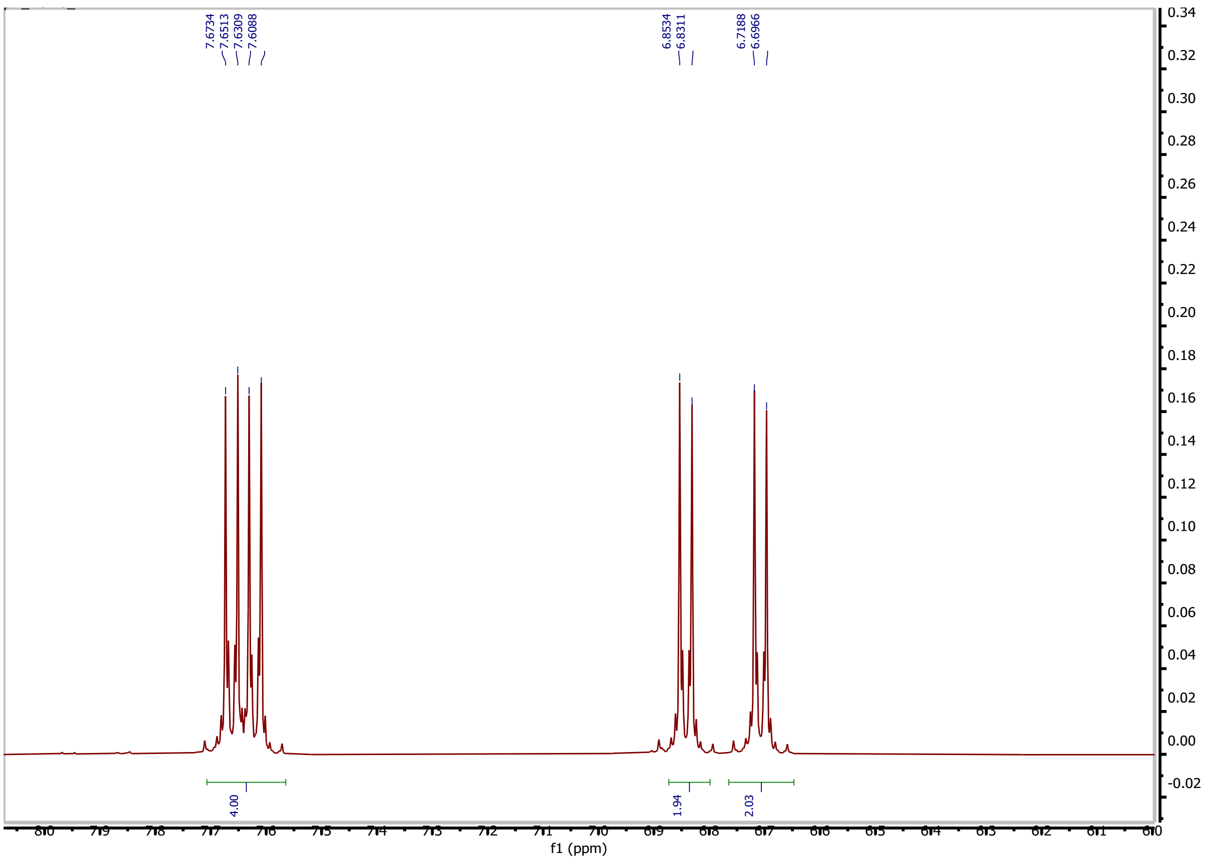

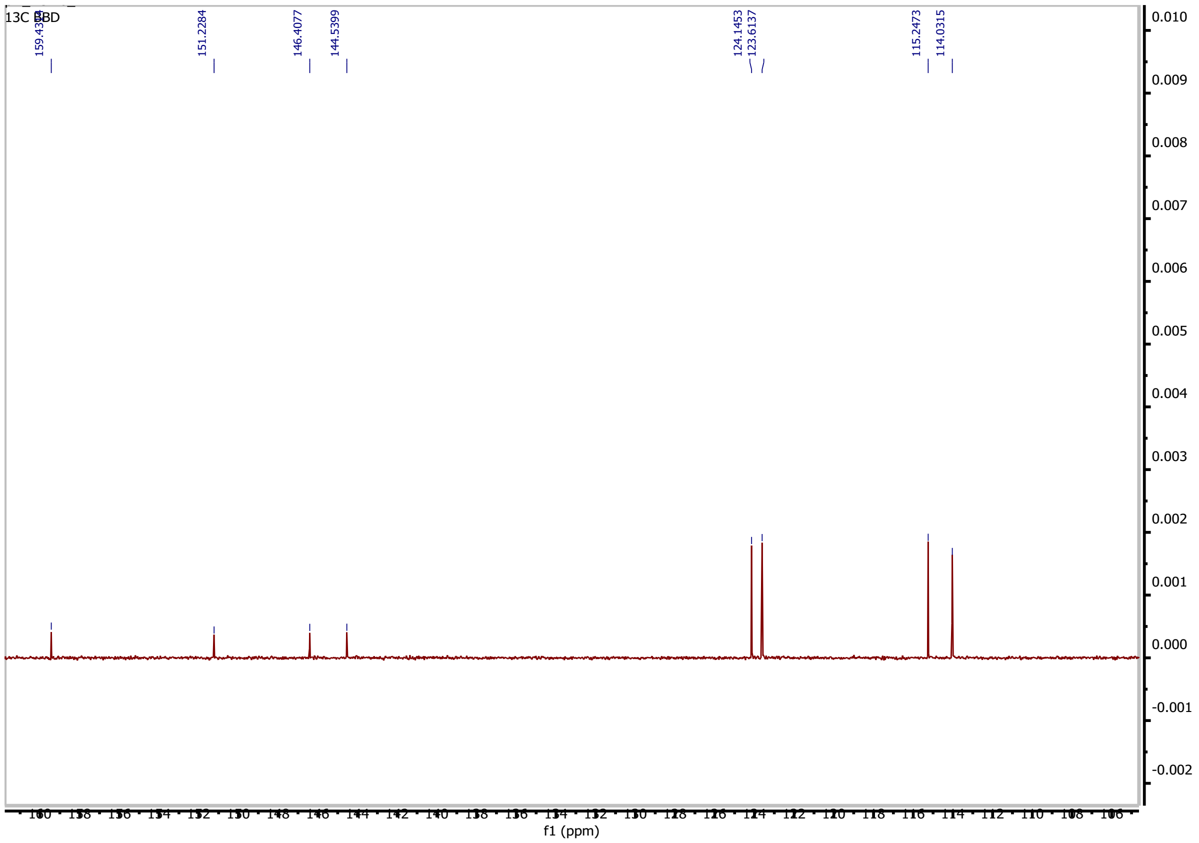

Figure S3. ^{1}H and ^{13}C-NMR spectra for compound OH–azo–NH_2 (CD_3OD).

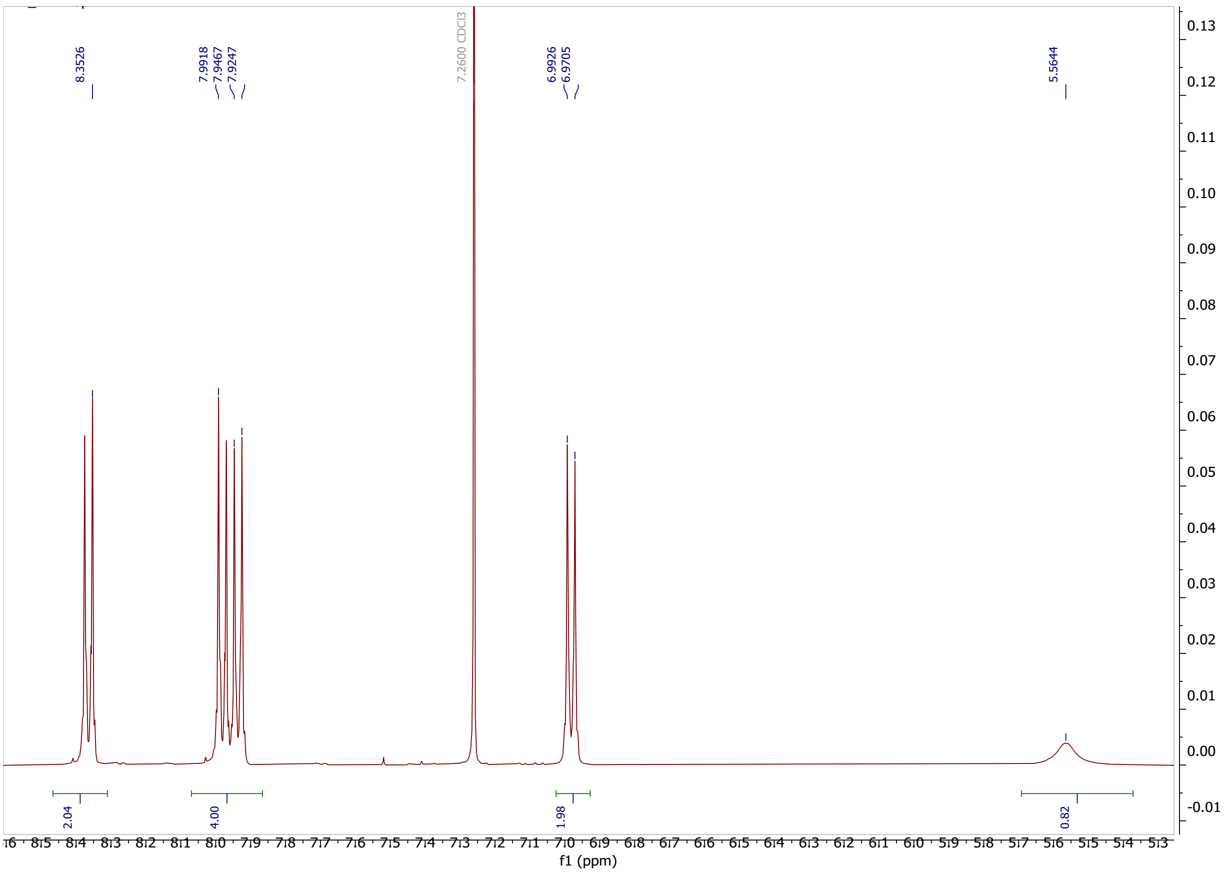

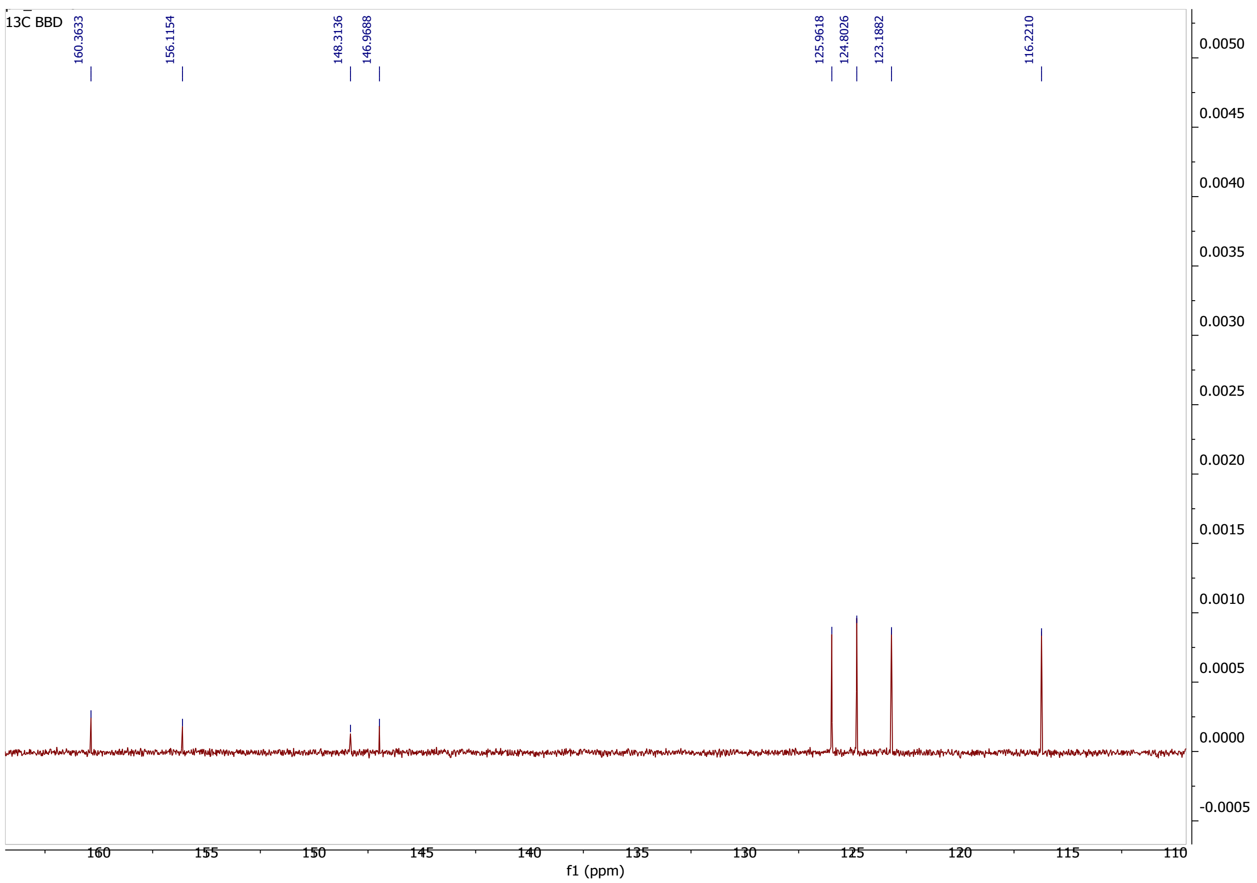

Figure S4. ^{1}H and ^{13}C-NMR spectra for compound OH–azo–NO_2 **(**$CDCl_3$).

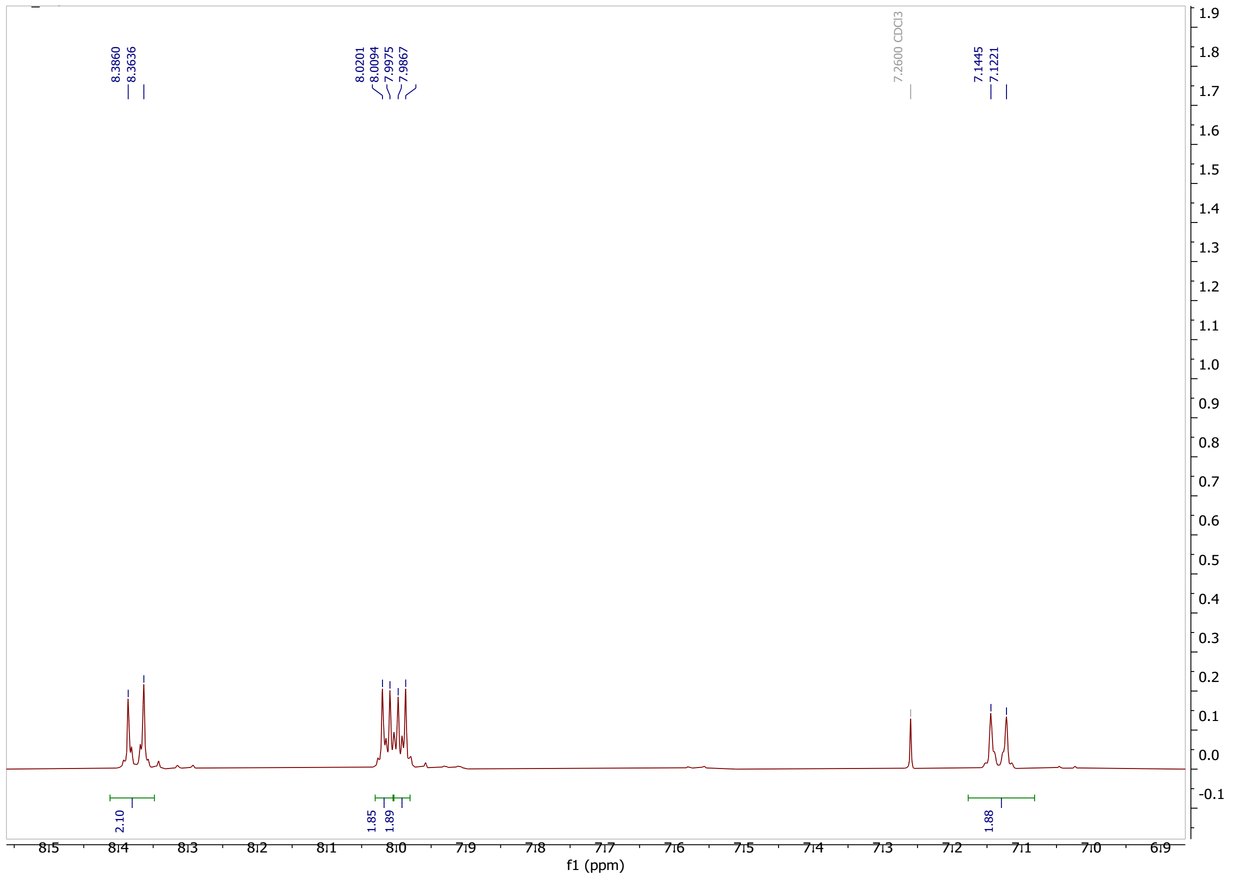

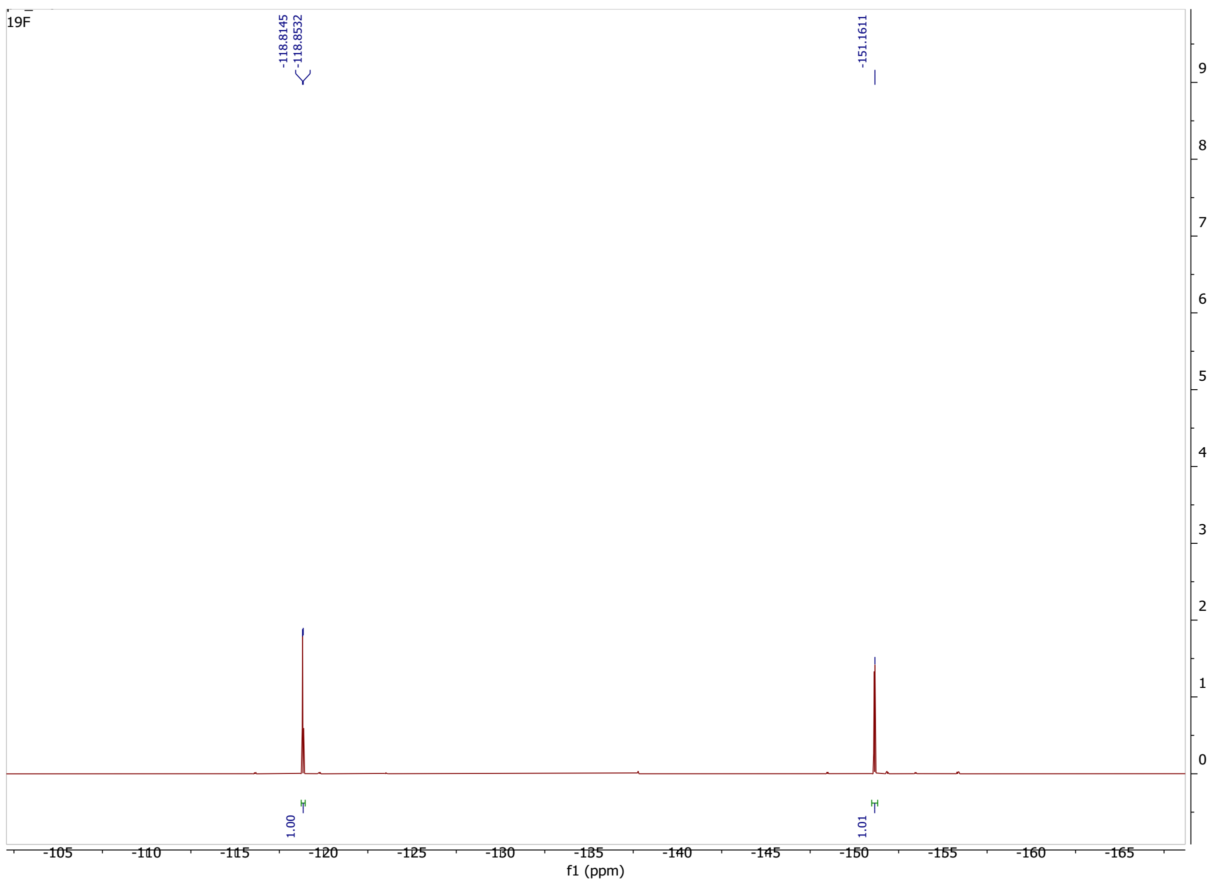

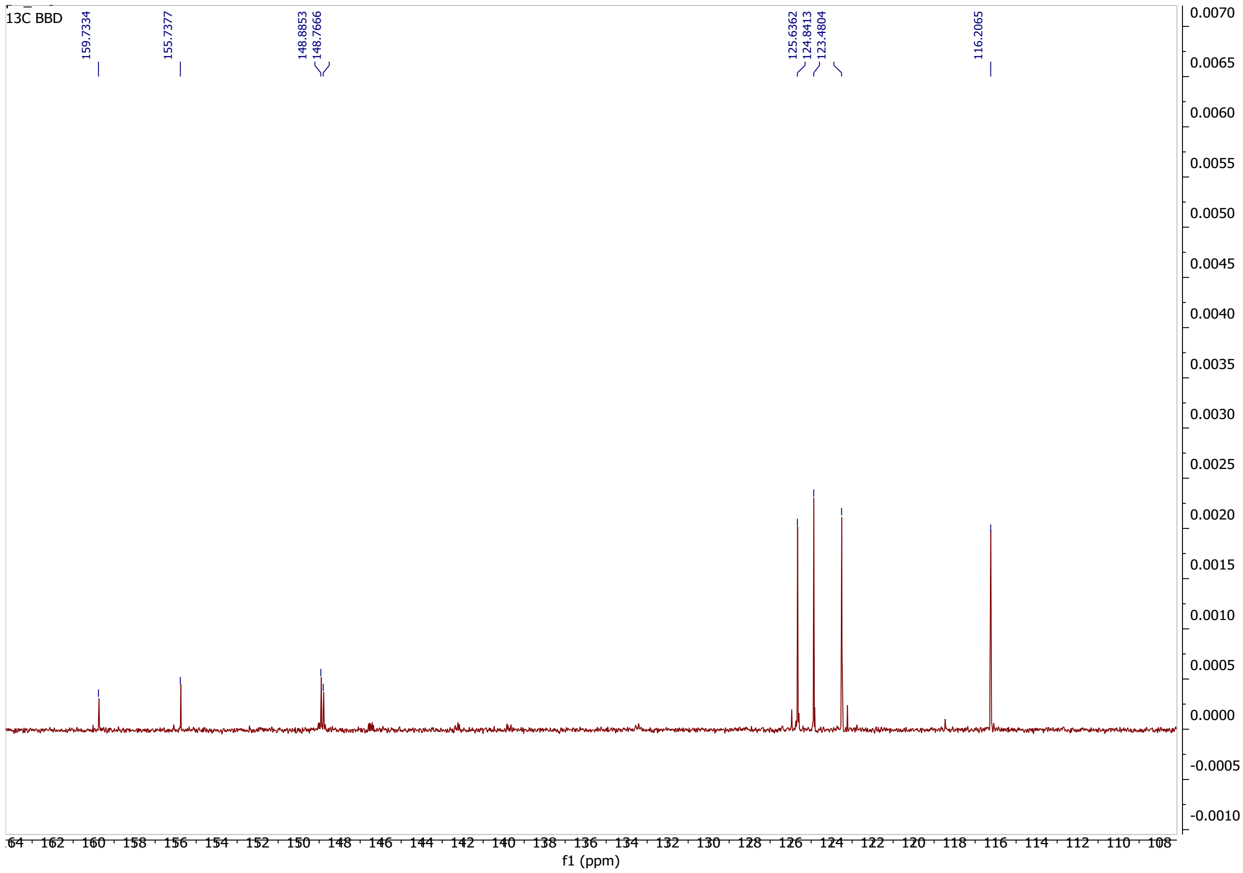

Figure S5. ^{1}H, ^{19}F and ^{13}C-NMR spectra for compound I–azo–NO_2 ($CDCl_3$).

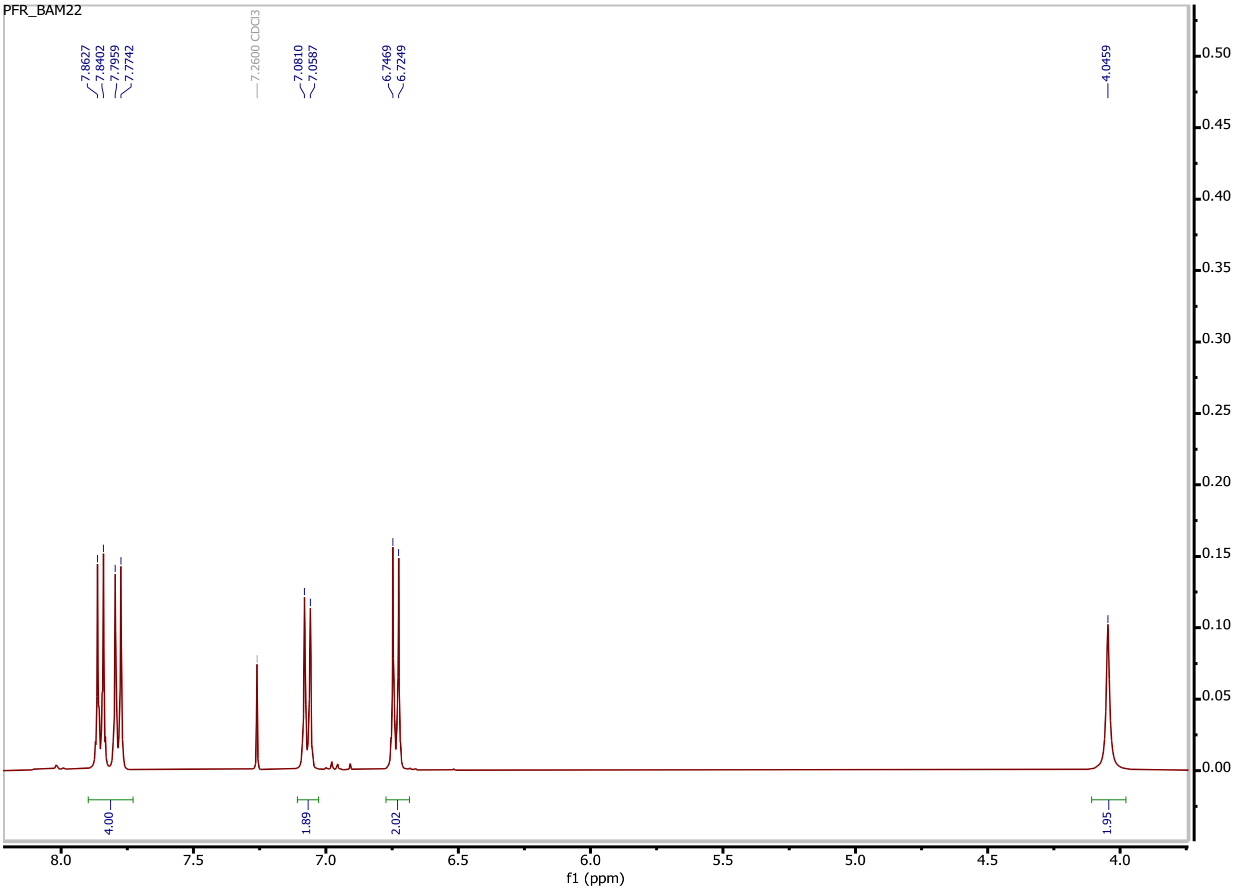
PFR_BAM22
7.8627
7.8402
7.7959
7.7742
7.2600 CDCl3
7.0810
7.0587
6.7469
6.7249
4.0459
4.00
1.89
2.02
1.95
f1 (ppm)

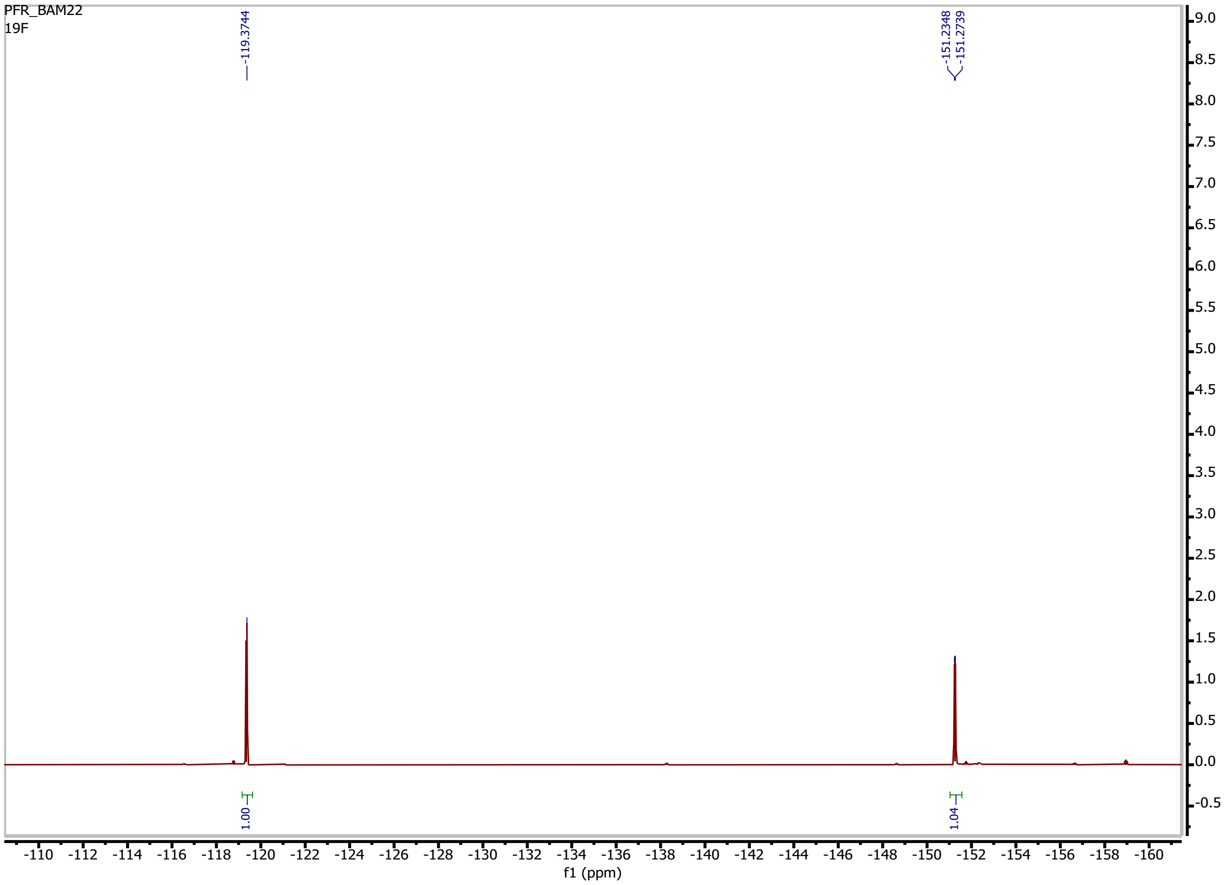
PFR_BAM22
19F
-119.3744
-151.2348
-151.2739
1.00
1.04
f1 (ppm)

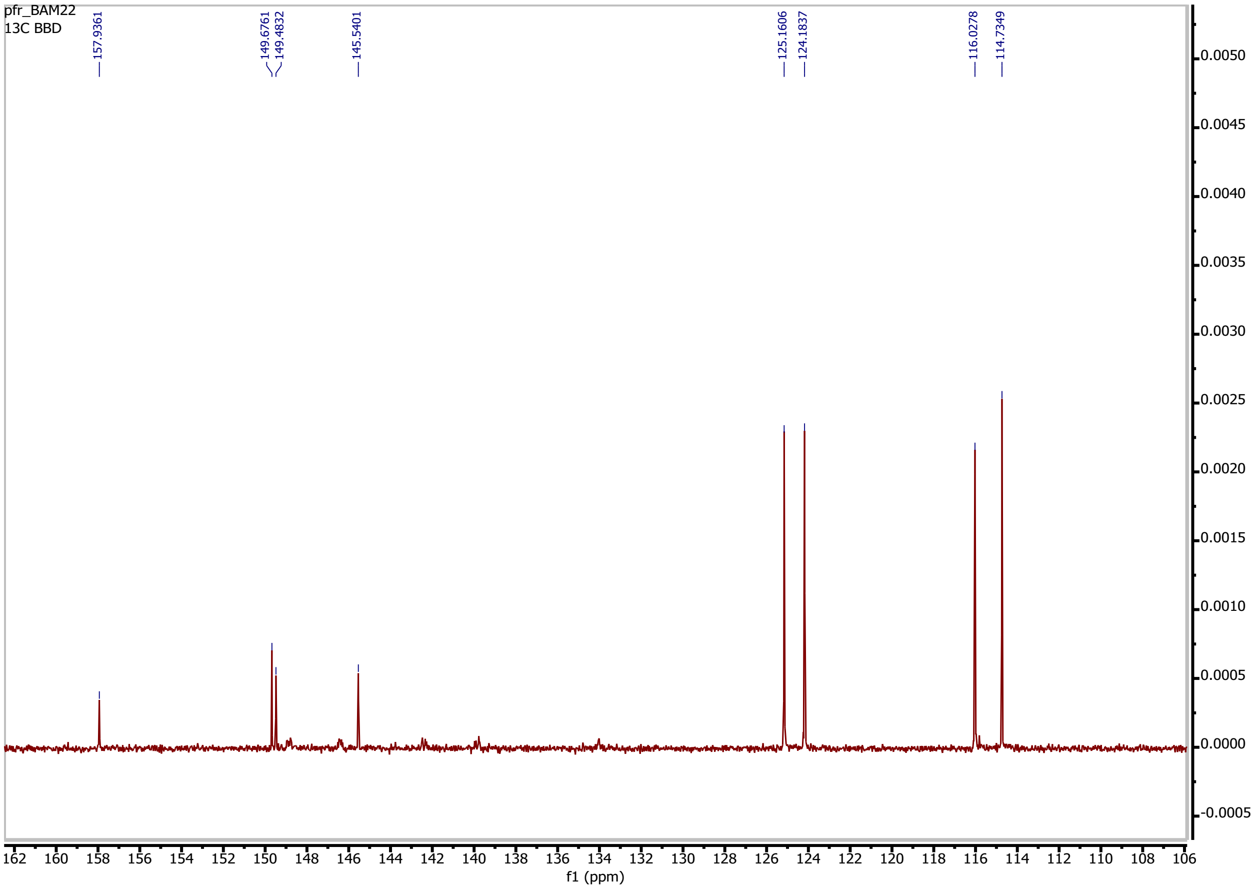

Figure S6. ^{1}H, ^{19}F and ^{13}C-NMR spectra for compound I–azo–NH_2 ($CDCl_3$).

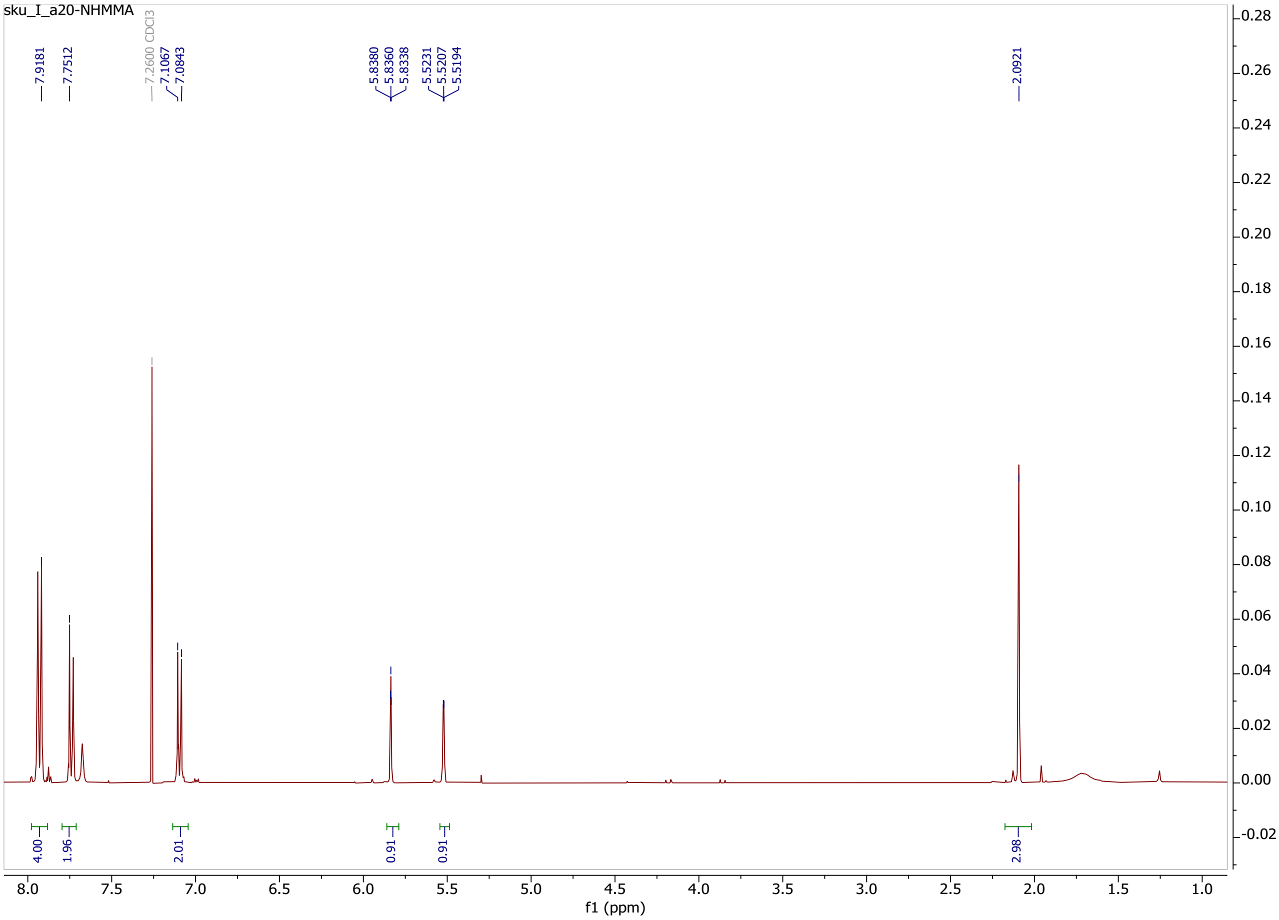
sku_I_a20-NHMMA
7.9181
7.7512
7.2600 CDCl3
7.1067
7.0843
5.8380
5.8360
5.8338
5.5231
5.5207
5.5194
2.0921
4.00
1.96
2.01
0.91
0.91
2.98
f1 (ppm)

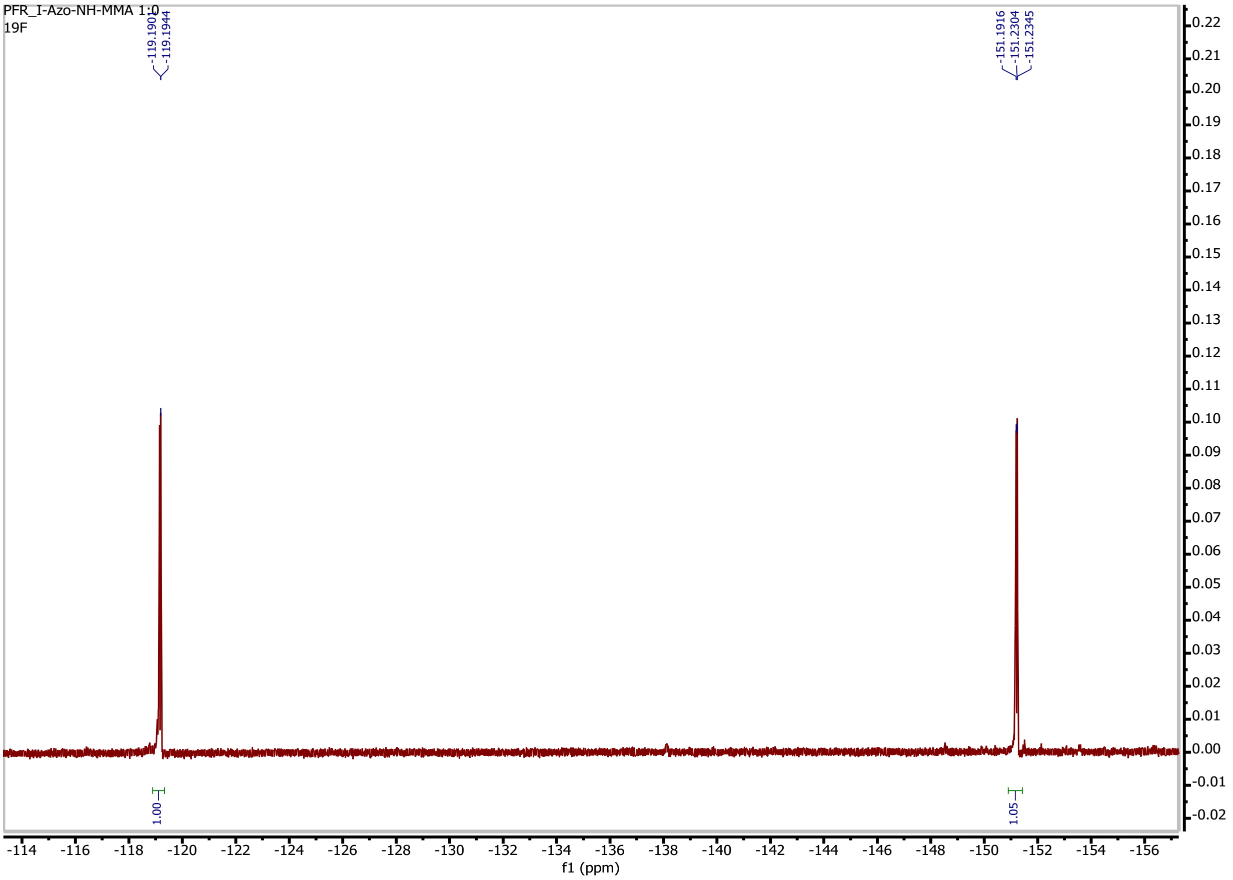
PFR_I-Azo-NH-MMA 1:0
19F
-119.1901
-119.1944
-151.1916
-151.2304
-151.2345
1.00
1.05
f1 (ppm)

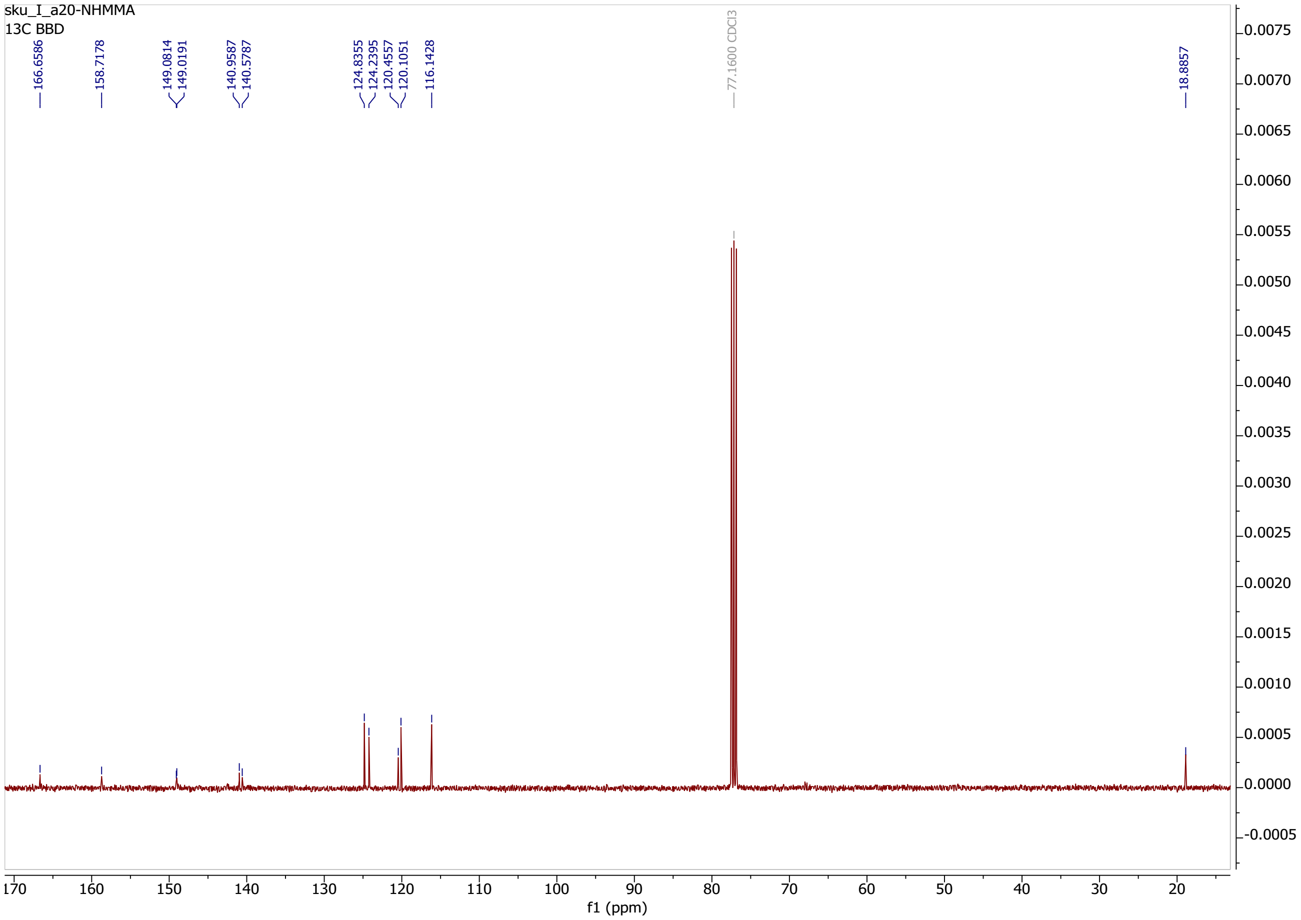

Figure S7. ^{1}H, ^{19}F and ^{13}C-NMR spectra for compound I–azo–NH–MMA ($CDCl_3$).

Scheme representing the self-complementary Ar-I···O_2N-Ar synthon

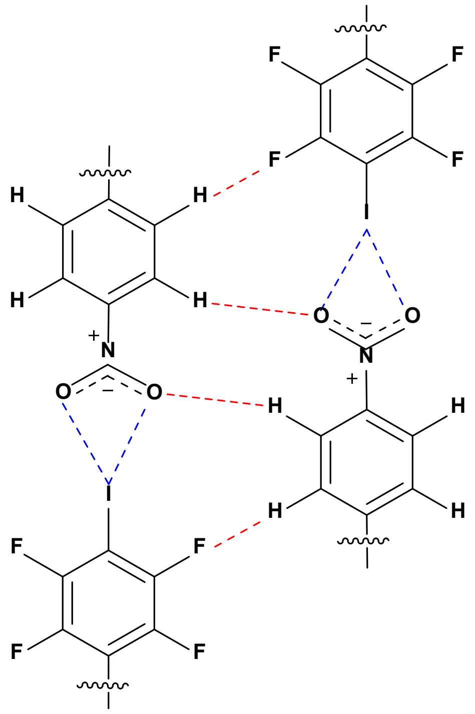

Scheme 1. Self-complementary Ar–I···O_2N–Ar synthon. Halogen and hydrogen bonds are represented in blue and red dashed lines, respectively.

NMR titration of azo dyes with tetrabutylammonium chloride

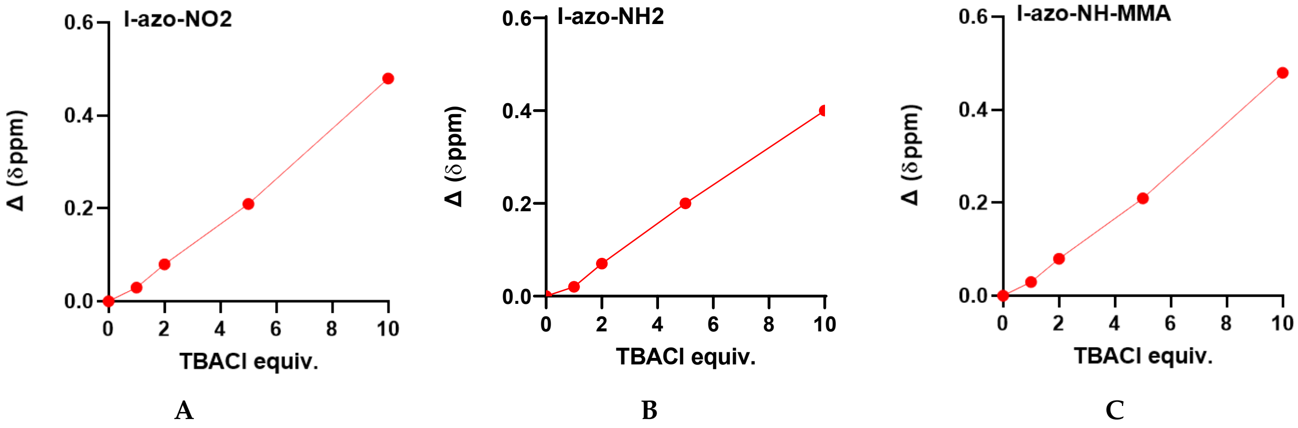

Figure S8. ^{19}F-NMR titration curves of I–azo–NO_2 (**A**), I–azo–NH_2 (**B**) and I–azo–NH–MMA (**C**) with tetrabutylammonium chloride. Molar ratios (azo dyes/TBACl): 1:0; 1:1; 1:2; 1:5; 1:10.
